# *Escherichia coli* O127 group 4 capsule proteins assemble at the outer membrane

**DOI:** 10.1371/journal.pone.0259900

**Published:** 2021-11-15

**Authors:** Matthew R. Larson, Kassia Biddle, Adam Gorman, Sarah Boutom, Ilan Rosenshine, Mark A. Saper

**Affiliations:** 1 Department of Biological Chemistry, University of Michigan, Ann Arbor, Michigan, United States of America; 2 Program in Biophysics, University of Michigan, Ann Arbor, Michigan, United States of America; 3 Dept of Microbiology and Molecular Genetics, Hebrew University Faculty of Medicine, Ein Kerem, Jerusalem, Israel; Centre National de la Recherche Scientifique, Aix-Marseille Université, FRANCE

## Abstract

Enteropathogenic *Escherichia coli* O127 is encapsulated by a protective layer of polysaccharide made of the same strain specific O-antigen as the serotype lipopolysaccharide. Seven genes encoding capsule export functions comprise the group 4 capsule (*gfc*) operon. Genes *gfcE*, *etk* and *etp* encode homologs of the group 1 capsule secretion system but the upstream *gfcABCD* genes encode unknown functions specific to group 4 capsule export. We have developed an expression system for the large-scale production of the outer membrane protein GfcD. Contrary to annotations, we find that GfcD is a non-acylated integral membrane protein. Circular dichroism spectroscopy, light-scattering data, and the HHomp server suggested that GfcD is a monomeric β-barrel with 26 β-strands and an internal globular domain. We identified a set of novel protein-protein interactions between GfcB, GfcC, and GfcD, both *in vivo* and *in vitro*, and quantified the binding properties with isothermal calorimetry and biolayer interferometry. GfcC and GfcB form a high-affinity heterodimer with a *K*_D_ near 100 nM. This heterodimer binds to GfcD (*K*_D_ = 28 μM) significantly better than either GfcB or GfcC alone. These *gfc* proteins may form a complex at the outer membrane for group 4 capsule secretion or for a yet unknown function.

## Introduction

Enteropathogenic and enterohemorrhagic *Escherichia coli* (EPEC and EHEC) cause severe intestinal infections such as the life-threatening acute diarrhea in infants, and hemorrhagic colitis, respectively [1]. Serotypes from both groups inject effector proteins through a type III protein secretion system into the cytoplasm of gut epithelial cells and restructure actin into “attaching and effacing lesions.” The effector protein Tir then becomes the receptor for attachment of EPEC. Other type III effectors further interfere with cell signaling pathways to disrupt intercellular contacts or inhibit inflammation and cell death pathways [2].

EPEC and EHEC are also encapsulated in extracellular polysaccharides and lipopolysaccharides (LPS) containing O-antigen. Capsules surrounding cells may act as virulence factors by shielding the bacteria from complement recognition [3, 4] or from phagocytosis [4], and by blocking binding of anti-microbial peptides to bacterial surfaces [3]. EPEC, EHEC, and *Shigella* serotypes, as well as other species, form a capsule from the same strain-specific O-antigen repeats found in their respective LPS [5–9]. As such, these are described as O-antigen capsules [5] and all belong to the group 4 capsule (*gfc*) biosynthetic pathway [10]. The observation that the O-antigen capsule of EHEC O157:H7 is produced and inversely regulated with the transcription of the typeIII secretion system suggested that the O-antigen capsule may shield the type III apparatus and intimin prior to cell attachment of EHEC [8]. O-antigen in capsules or attached to LPS was shown to protect EPEC O127 from human alpha-defensin 5 [11].

Biosynthesis of the O-antigen oligosaccharides for capsule and LPS is encoded at the O-antigen locus. The oligosaccharide repeat attached to the lipid carrier undecaprenyl pyrophosphate is synthesized in the cytoplasm [12] and then the O-antigen unit transporter Wzx flips the O-antigen repeat across the inner membrane leaflet. Wzy then elongates the O-antigen polymers in the periplasm by transferring new O-antigen units (a tetrasaccharide for *E*. *coli* O127) to the reducing terminus of the lipid-linked polysaccharide [13]. The chain length of the LPS O-antigen polysaccharide is determined by Wzz and is typically 15–25 units but capsular polysaccharide chains may be much longer. O-antigen polysaccharides in LPS are linked to the core oligosaccharide by the WaaL ligase but O-antigen capsule synthesis and export may be independent of WaaL [11].

**Fig 1:**
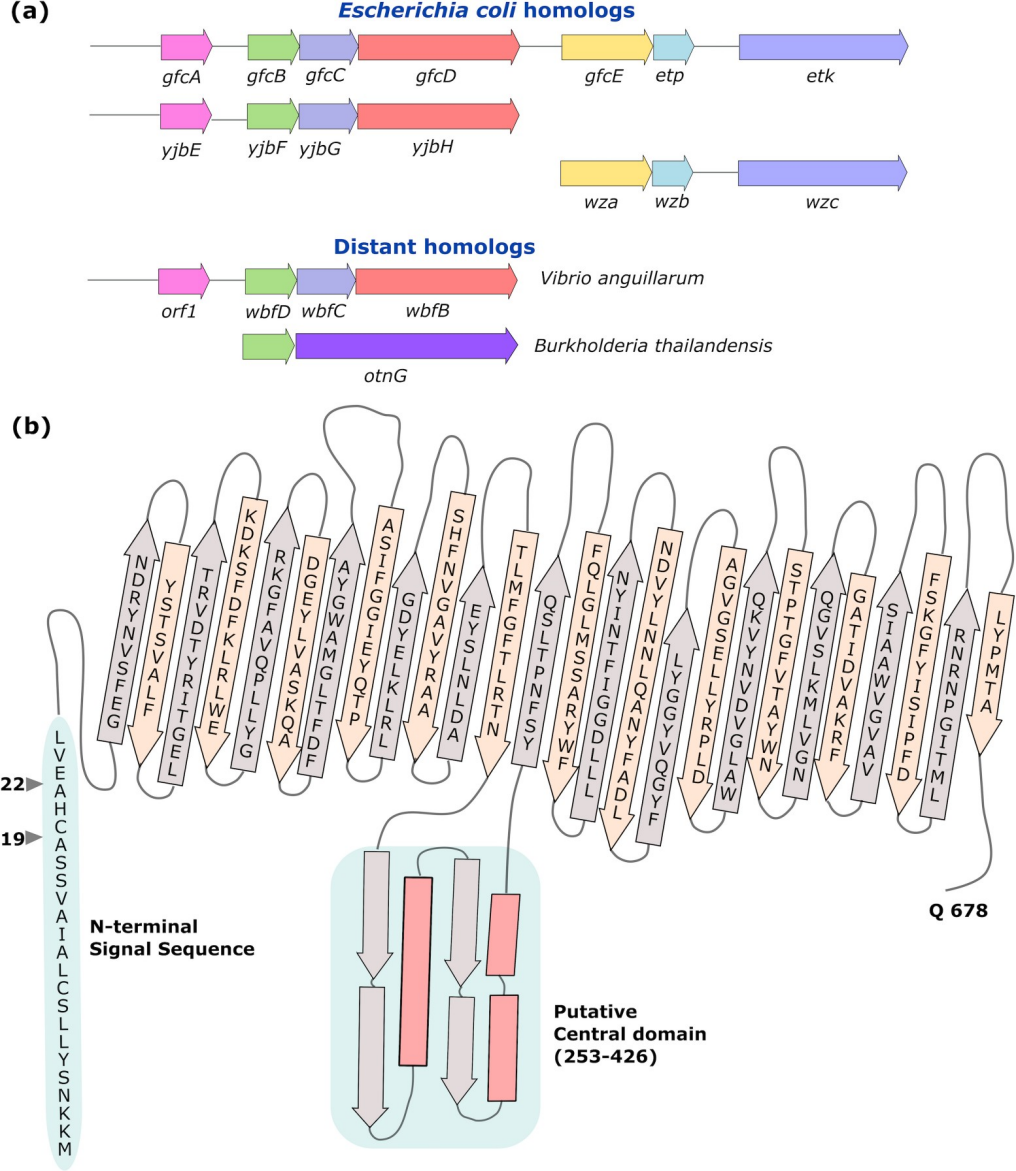
Group four capsule operon and GfcD. **(a)** The *Escherichia coli* group 4 operon consists of seven genes. The genes *gfcA*, *gfcB*, *gfcC*, *and gfcD* encode proteins of an unknown function. Genes *gfcE*, *etp*, *and etk* share homology with proteins of Group 1 capsule export systems. Distant homologs are also found in *Vibrio and Burkholderia sp*ecies. **(b)** Membrane topology of GfcD as predicted by HHOMP and classified as a potential outer membrane β-barrel protein. The central region (273–427) is devoid of transmembrane strands and may be an independently folded domain with the predicted secondary structure shown. The signal sequence at the N-terminus (shaded blue) shows a putative signal peptidase II cleavage and acylation site (arrow 19) as previously annotated, and the peptidase I cleavage site (arrow 22) revealed in the present work by MALDI-TOF and N-terminal sequencing.

The second genetic locus encodes proteins necessary for exporting and possibly anchoring the O-antigen capsule. A gene encoding an inner membrane tyrosine kinase, *etk*, was first identified in this region [14], and later found to be a member of an operon containing seven genes subsequently named “*gfc*” for *g*roup *f*our *c*apsule [7] (Fig 1A). The *gfc* operon is present in EPEC, EHEC, enterotoxigenic *E*. *coli*, and select *Shigella* species. The three genes at the 3´ end of the operon (*gfcE*, *etp*, *etk*) are paralogs of *wza*, *wzb*, and *wzc* located in operons encoding colanic acid or other group 1 capsules. Upstream of the *gfcE* gene, but encoded on the same mRNA transcript [7], are four genes *gfcA*, *gfcB*, *gfcC*, and *gfcD* that are without definitive functions.

It is known that *etp* and *etk* encode tyrosine phosphatase and kinase enzymes, respectively, that drive O-antigen polysaccharide export, presumably by the cycling of phosphorylation and dephosphorylation of Etk [15, 16]. Etk is likely an octamer based on the crystal structure of the nonphosphorylated form of Wzc [17]. Based upon its high sequence identity with *wza*, *gfcE* encodes a putative export portal. In the group 1 capsule system, the homologous Wza forms an octamer in the outer membrane to form a pore 15 Å in diameter [18] and the exit portal for group 1 capsules [19]. The periplasmic domains of Wzc and Wza interact to form a conduit spanning the two membranes [20]. It was previously reported that loss of any of the *gfc* genes disrupted the buoyancy phenotype associated with the EPEC O127 O-antigen capsule [7]. Similarly, deletion of the *gfc* operon in *Shigella sonnei* resulted in loss of the O-antigen capsule [9]. The anchoring of high molecular weight O-antigen polysaccharide capsules distinct from LPS could occur at cell surface attachments to lectin-like protein receptors such as the example set by the group 1 protein Wzi [21, 22].

The set of genes *gfcABCD* with yet unknown function has homologs in *E*. *coli* and more distant species (Fig 1A). Current evidence indicates *gfcABCD* and homologous operons of other species—*Vibrio cholera* O139 *wbfDCB* [23], *Vibrio anguillarum wbfDCB* [24], *Aeromonas hydrophila gfcABCD* [25] and the *E*. *coli yjbEFGH* [26]—have functions linked to capsule or exopolysaccharide production. *E*. *coli gfcA*, the shortest gene, has an unusually rich percentage of threonine residues (30%) and may encode a 101-amino acid inner membrane or secreted protein. The *gfcB* gene was identified in a screen of *E*. *coli* O157:H7 gene disruptions that impaired colonization of a bovine host [27] suggesting that GfcB is involved in virulence. GfcB, GfcC, and GfcD all contain predicted signal sequences for export to the periplasm. The signal sequences of GfcB and GfcD also contain lipobox motifs including cysteine residue(s) that would becleaved with signal peptidase II, modified with acyl groups, and localized to the cell outer membrane.

GfcD is annotated as a putative lipoprotein in many of the online sequence databases, but an alternative site for signal peptidase 1 cleavage was predicted by SignalP 4.0 [28] indicative of a non-lipidated protein. Further, GfcD is predicted with high likelihood to belong to the cluster OMP.nn.30.1 of outer membrane β-barrels in the HHOMP database [29].

Our lab has pursued structural studies of these *gfc* proteins to decipher the function of *gfc* and its widespread homologs. The crystal structure of GfcC has revealed fold similarities to Wza [30]. These include the presence of two β-grasp domains in GfcC like the periplasmic domains of Wza. GfcC, however, is a soluble periplasmic protein and it is not clear yet what functional role it has in capsule export. The crystal structure of GfcB (previously called YmcC, PDB id 2IN5, unpublished) shows a homodimer of a polypeptide containing an 11-stranded β-sandwich.

In this paper, we describe our effort to over-express and purify the integral membrane protein GfcD. We find that GfcD is indeed an outer membrane protein but not a lipoprotein. Within EPEC O127, the GfcB, GfcC, and GfcD interact at the *E*. *coli* cell envelope and we identified a complex containing all three proteins. Subcomponents GfcB and GfcC associated with high affinity to form a stable protein heterodimeric complex. These studies are the first to characterize the complex interactions of the *gfc* proteins at the *E*. *coli* outer membrane.

## Results

### β-barrel topology prediction for GfcD

The *gfcD* gene is the fourth coding region in the *gfc* operon and overlaps the 3´ end of *gfcC* (Fig 1A). The encoded 698-amino acid polypeptide GfcD has a high proportion of tyrosine (6.4%) residues as is often found in outer membrane β-barrel proteins. The algorithm of HHOMP performs Hidden Markov model pairwise alignments to an underlying database of more than 20,000 putative outer membrane protein sequences [29]. HHOMP predicts a 96.8% overall probability that GfcD is an outer membrane protein and within cluster 116. The annotated secondary structure for the sequences gives 26 transmembrane strands with a potential central domain region separate from the barrel topology (Fig 1B). The large central segment from amino acid residues 277–425 is predicted to lack transmembrane strands and, based on secondary structure analysis, was predicted to contain 3–4 α-helical segments and an equal number of short β-strands, potentially folding into a likely periplasmic α/β domain (Fig 1B). See **Discussion** for an alternative, multi-barrel topology for GfcD.

### Over-expression of GfcD protein

GfcD protein over-expressed from pSA10(*gfcD*) in BL21(DE3) produced insoluble inclusion bodies, but post-induction yielded 100-fold fewer colonies on antibiotic containing vs non-antibiotic LB agar plates. Strains transformed with pSA10(*gfcD*) with leaky basal expression [31] grew poorly on plates with IPTG compared to the empty pSA10 controls. Observations of poor growth and plasmid loss are common with toxic protein expression [32]. We reduced toxic effects in two ways: we inserted *gfcD* into plasmids (pMCSG26 and pBH31) containing T7 promoters with tighter control on expression and higher overall induction, and also transformed the plasmid into the C41(DE3) strain of *E*. *coli* which has been shown to better express membrane proteins [33]. However, when we compared the C41(DE3) strain with a Tuner^TM^(DE3)/pLacI system, the Tuner^TM^(DE3)/pLacI cells produced a higher yield of GfcD (> 3 mg L^–1^) than did C41(DE3) (typically 1 mg L^–1^ or less). Yields were highest when the cell culture was induced after cell density reached the OD_600_ of 2.0. In summary, specific host cell and expression conditions limited apparent toxic effects of over-expressing GfcD.

**Fig 2:**
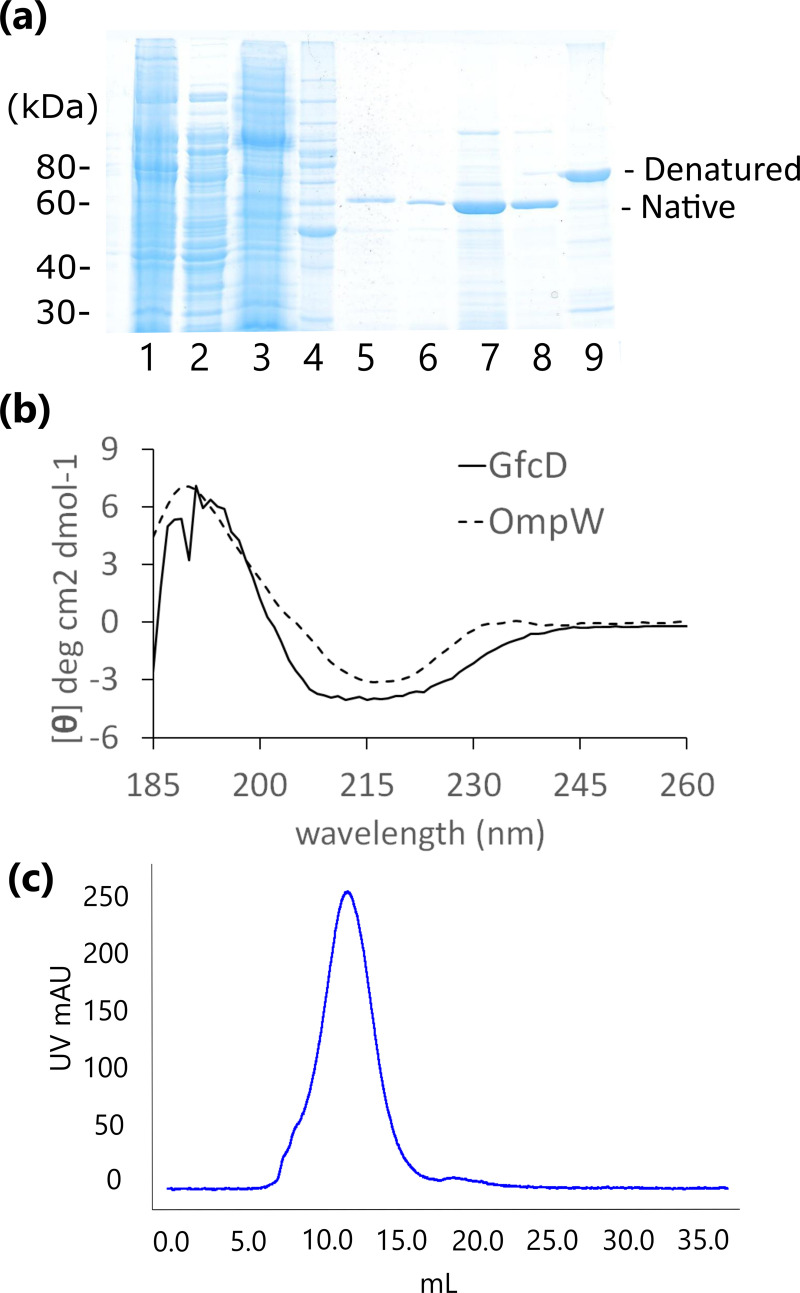
Purification of GfcD. (a) SDS-PAGE gel showing the purification of GfcD, encoded from pBH31 vector. The lysates (lane 1) were separated by ultracentrifugation into soluble (lane 2) and membrane (lane 3) fractions. Lane 4 is the molecular weight ladder (Invitrogen Benchmark). After solubilizing GfcD with 5% Elugent, it was affinity purified on TALON (lane 5). His-tag was removed by TEV cleavage (lane 6), and then GfcD was purified by ionic exchange (lane 7) followed by size exclusion purification (lane 8). Boiling the sample results in a migration shift (native vs. denatured) common to β-barrel proteins upon unfolding (lane 9). (b) Circular dichroism spectra of GfcD protein compared against the known β-barrel protein OmpW. (c) The GfcD protein was exchanged into a buffer of 20mM Hepes pH 7, 150mM NaCl, 0.02% DDM over a Superdex 200 10/300 size exclusion column and demonstrated a symmetrical peak.

Minor contaminants were problematic during protein purification (Fig 2A), including a chromophore of reddish color identified as ubiquinol oxidase by mass spectroscopy that required the addition of the reducing agent TCEP to separate it from GfcD fractions. Rapid protein degradation occurred when GfcD was exchanged into some detergents including *N*,*N*-dimethyldodecylamine N-oxide (LDAO), octyl glucoside (OG), or nonyl-glucoside (NG). GfcD was stable in decyl-maltoside (DM) or *n*-dodecyl-β-D-maltopyranoside (DDM) where it could be kept at 37°C for 24 hours without degradation. DDM has a reported micelle size of 74 kDa while LDAO micelles are smaller at 17 kDa [34]. GfcD stability in detergent was correlated with the size of the detergent micelles where LDAO and other smaller micelle detergents promoted rapid degradation of GfcD into two 30–35 kDa fragments. Our speculation is that larger micelles may restrict access to loop regions that contain cleavable sites for proteases.

### GfcD is not a lipoprotein

GfcD is annotated in databases such as UniProtKB [35] as a putative lipoprotein. The highest probability acylation site predicted by LipoP [36] is Cys19. Meanwhile, LipoP and SignalP 4.0 [28] give a higher likelihood for a signal peptidase I cleavage site (non-lipoprotein) between residues Ala21 and Glu22. To locate an amino-terminal peptide with modifications including acylation, purified GfcD was subjected to an in-gel tryptic/chymotryptic digest followed by MALDI-TOF (S1 Fig). No peptide matches with weights for an acylated N-terminal peptide fragment were identified and we detected an amino-terminal peptide fragment beginning at Glu22, *i*.*e*. the predicted cleavage site for the type I signal peptidase. Edman degradation sequencing of purified GfcD confirmed the first 6 residues as EVLTP (E22–P27). We concluded that recombinant GfcD is not a lipoprotein as annotated in the protein sequence databases. Knowing that modification of the N-terminus occurs via signal peptidase I, we focused on expressing GfcD from pBH31 (S1 Table) that encodes the *pelB* signal sequence followed by His_10_ tag, TEV protease site, and mature sequence of GfcD (residues 22–698).

### GfcD is mostly comprised of β-strand

Circular dichroism spectra were collected for GfcD and compared against the known outer membrane protein OmpW (Fig 2B). Spectra were deconvoluted by SOMCD [37] to get secondary structure estimations (Table 1). The circular dichroism spectra of GfcD support a model with overall β-barrel conformation as seen by the large percentage of β-strand conformation in the HHOMP-derived topology model (Fig 1B). Helical content from SOMCD is 16.2% compared to 10.6% predicted by HHOMP. This likely reflects the helical content of the inserted domain discussed above. We also observed that GfcD displayed heat-dependent migration shifts on SDS-PAGE gels [Fig 2A, lanes 8 (native) & 9 (denatured)], a property described as “heat-modifiability” that has been commonly seen with outer membrane β-barrels [38]. Un-boiled samples of GfcD ran at a smaller than expected size of 60 kDa on SDS-PAGE, while boiled samples ran as the expected 76.6 kDa size (based on the amino acid sequence).

**Table 1:** Secondary structure of GfcD.

Source	α-helical	β-strand	Turn	Random
Circular Dichroism	16.2 ± 7.4%	39.4 ± 4.8%	9.7 ± 1.0%	34.7 ± 5.0%
HHOMP consensus for outer membrane protein cluster 116.	10.6% (74 residues)	42.2% (295 residues)	5.6% (39 residues in periplasmic turns)	41.5% (290 residues no secondary structure)

### GfcD is localized to the outer membrane

**Fig 3:**
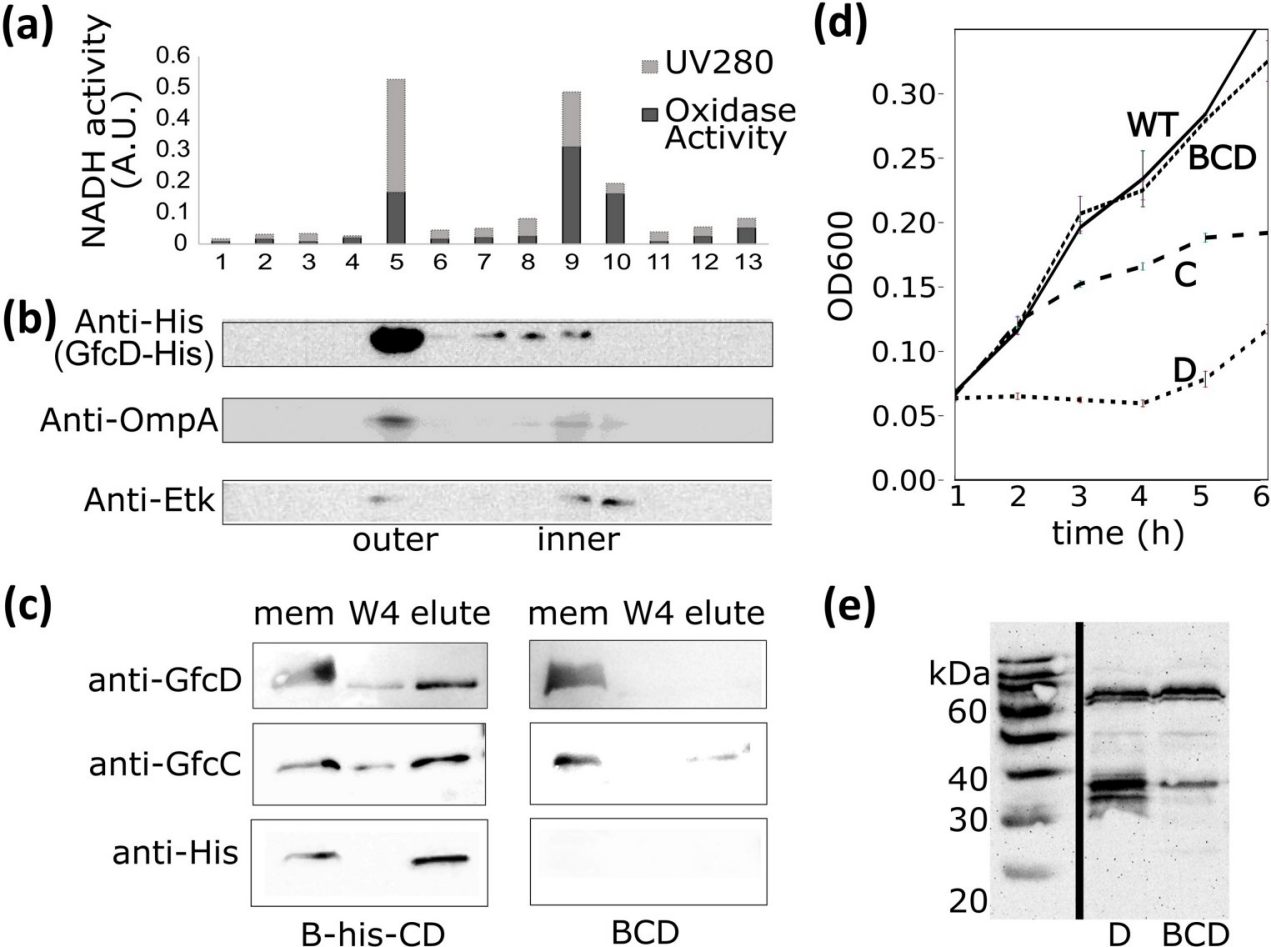
Localization and protein interactions of GfcD. GfcD-6His dominantly localizes to outer membrane fractions. **(a)** NADH oxidase activity was measured for each fraction and **(b)** fractions were blotted for known markers of the inner (Etk) and outer membrane (OmpA) and the His-tag of GfcD to define inner and outer membrane fractions (original blot scans in S1 Raw images). **(c)** GfcB-His6, GfcC, and GfcD were co-expressed in EPEC cells and pulled-down with NiNTA beads. GfcC and GfcD proteins were detected on Western blots. *Right*, Absence of a His-tagged GfcB results in loss of pull-down of GfcC or GfcD. Abbreviations: **Mem**: membrane fraction; **W4**: fourth wash of pull-down, **Elute:** pull-down product. **(d)** Over-expressing GfcC or GfcD alone inhibits cell growth of EPEC; no inhibition is observed during co-expression of GfcB, GfcC, and GfcD (**BCD**). **(e)** Anti-GfcD blot comparing levels of GfcD when expressed alone or with GfcB and GfcC. Full length GfcD is at 60 kDa on this blot. GfcD rapidly undergoes proteolysis when expressed alone.

GfcD was found in cellular membrane fractions of *E*. *coli* after lysis. We fractionated inner and outer membrane leaflets of EPEC over a sucrose gradient to identify to which membrane layer GfcD localizes. The activity of NADH oxidase (Fig 3A) defined the inner membrane fractions. Blotting for the inner membrane Etk protein and the outer membrane OmpA further defined the inner and outer membrane fractions, and we detected the majority of GfcD in the outer membrane (Fig 3B).

### GfcD is monomeric

In order to characterize the stability of GfcD by its UV absorption profile during elution from a size exclusion column, GfcD expressed from pBH31 was exchanged into other detergents, such as LDAO, DG, or DDM, by passage through a Superdex 200 10/300 GL column. GfcD had a symmetrical peak in DDM while in DG or LDAO it exhibited asymmetric and multiple peaks. We chose DDM to maintain stability of GfcD based on the proteolytic stability and symmetrical elution profiles in the detergent (Fig 2C). The oligomeric state of the GfcD protein in the DDM detergent was determined by multi-angle light scattering (SEC-MALS, S2 Fig). The first eluted peak corresponds to the GfcD:DDM complex while the second peak are the detergent micelles alone with negligible amounts of protein. SEC-MALS with protein-conjugate analysis determined that peak 1 contains the DDM detergent with mass of 158 kDa and protein mass of 76.6 kDa, consistent with a monomer (S3 Table).

### GfcD interacts with GfcB and GfcC

The reading frames for the *gfcB*, *gfcC*, and *gfcD* genes overlap by 1–4 base pairs. Homologous operons conserve this set of three adjacent genes suggesting that all three proteins function as a single unit. GfcD was localized to the outer membrane as shown above, while GfcB and GfcC are predicted to be an outer membrane lipoprotein and periplasmic protein [7], respectively. We tested whether they interact by co-expressing *gfcBCD* in EPEC and using a pull-down assay with GfcB as the bait protein to identify interactions. His-tagged GfcB pulled down both GfcC and GfcD proteins and the absence of tagged GfcB protein gave no pull-down of GfcC or GfcD (Fig 3C).

During expressions of GfcD we noticed limited yields, and suspected the known problem of toxicity with overexpression of membrane proteins. Individual over-expression of pSA10(*gfcC)* or pSA10(*gfcD)* in wild type EPEC negatively affected cell growth compared with EPEC with an empty pSA10 vector (Fig 3D). Yet when all three *gfcB*, *gfcC*, and *gfcD* genes were co-expressed in EPEC, the cell growth rates matched wild type. When GfcD was over-expressed alone it had increased proteolytic degradation and inhibited cellular growth (Fig 3E) compared to co-expression with GfcB/GfcC. GfcB and GfcC may promote the stability of GfcD or resistance to proteolysis, supporting the observations that all three proteins are functioning together at the outer membrane.

### A high-affinity interaction between GfcB and GfcC

**Fig 4:**
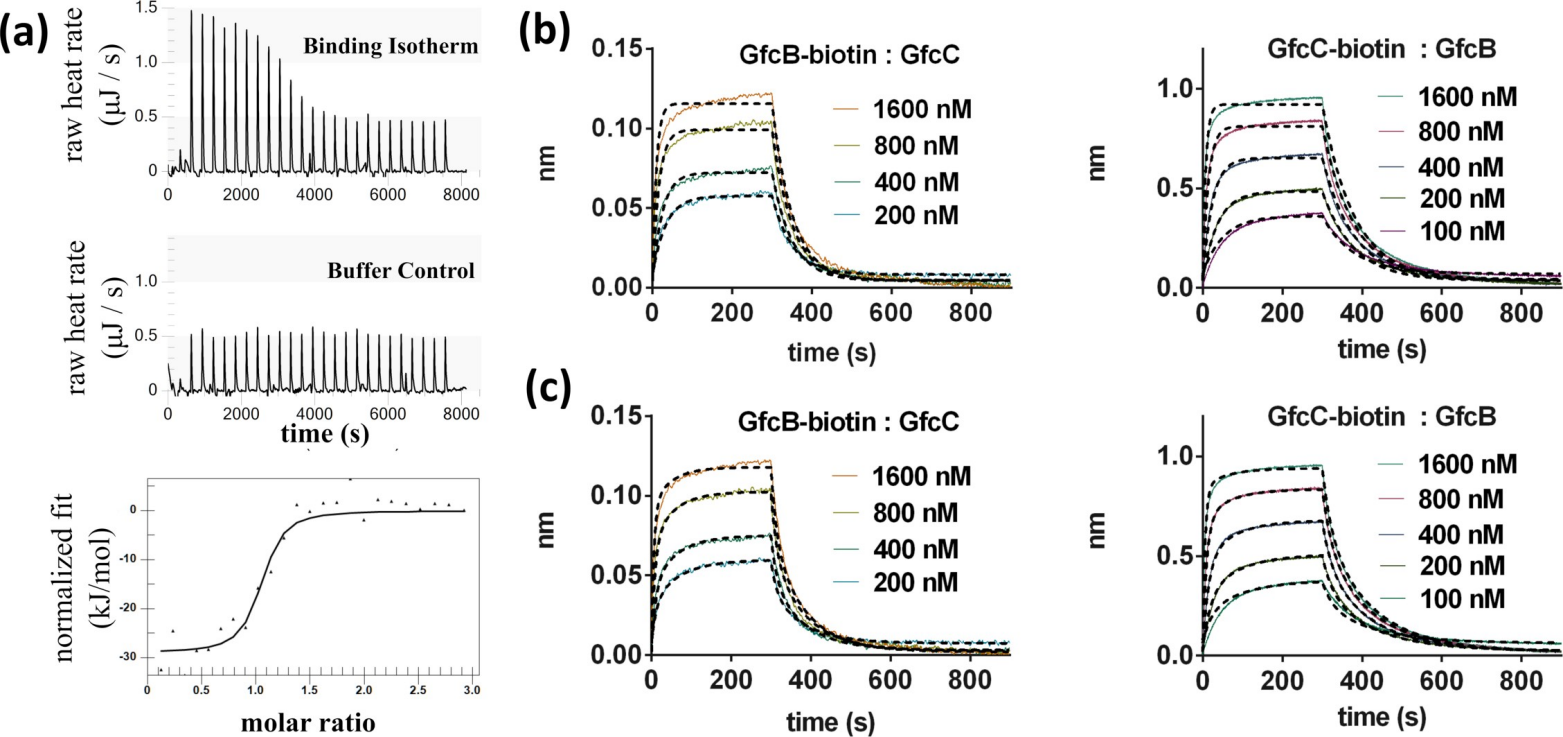
GfcB and GfcC form a tight complex. **(a)** Isothermal calorimetry. 24 injections of GfcB into GfcC at 25°C were measured (*upper*) and the heat released (μJ) at each injection was subtracted by the heats released for the control injections in buffer (middle) and were fit (*lower*). GfcB-GfcC have a 1:1 interaction with K_D_ of 0.1 μM in solution. (**b**) Biotinylated GfcB was anchored to an SA (streptavidin) sensor and the real-time binding response of GfcC at concentrations of 200 nM to 1.6 μM were measured by BLI. (**c**) Biotinylated GfcC on the sensor and binding of GfcB (with C-terminal His_6_-tag expressed from pET-BLUE2) at concentrations of 100 nM to 1.6 μM were measured. The BLI data in (**b**) and (**c**) were fit with either a one-site binding model (**top**) or one-site binding followed by conformation change model (**bottom**).

We measured the binding of GfcB and GfcC proteins at 25°C with isothermal titration calorimetry (ITC) to quantify binding affinities. The binding fit with near 1:1 stoichiometry of a simple heterodimer and was enthalpically driven (Δ*H* of –28.95 kJ mol^–1^) (Fig 4A). We observed a positive change in entropy (Δ*S* of 36.94 J K^–1^ mol^–1^) that may reflect a loss of solvation at the protein-protein interface or a conformational change upon binding of the proteins. There is tight binding between GfcB-GfcC in a one-site model where the equilibrium dissociation constant *K*_D_ is 99.8 nM (Table 2, top). The binding interaction of GfcB and GfcC was also studied with biolayer interferometry (BLI), with either protein immobilized to a streptavidin (SA) sensor and its partner free in solution. Non-specific interactions were minimized with the addition of 0.1 mg mL^–1^ bovine serum albumin in the buffers. Experimental signals were subtracted with the signal from an unlabeled control tip to remove any non-specific binding effects. With GfcC on the SA tip and GfcB [expressed from pETBlue2(*gfcB*)] in solution, the binding was fit to a one-site model (Fig 4B) and the measured equilibrium dissociation constant was 175 nM, or approximately 2-fold higher than ITC. When the binding was reversed, with GfcB [from pMCSG7(*gfcB*)] on the SA sensor, the *ka*_*1*_ *(kon)* rate was decreased and the *kd*_*1*_ (*koff)* rate increased for an overall 4-fold lower affinity (*K*_*D*_) of 374 nM (Table 2, bottom). This decrease in affinity when GfcB was on the SA tip was observed in multiple experiments and might be due to steric interference of the biotin labels present on the proteins for the BLI experiment. The binding kinetics had the best fit with a simple one-site model with conformational change (Fig 4C), and models with or without conformational change gave similar fits to the *ka*_*1*_ (*kon*) and *kd*_*1*_ (*koff*) rate kinetics (Table 2, bottom).

**Table 2:** Affinities of interactions of GfcB, GfcC, and GfcD.

**Isothermal calorimetry**	**Model**	**Δ*G* (kJ/mol)**	**Δ*H* (kJ/mol)**	**Δ*S* (J K**^**–1**^ **mol**^**–1**^**)**	***K***_***D***_ **(nM)**	***n***	
GfcB, GfcC	1:1	–39.96	–28.95	36.94	99.8	1.013	
GfcBC, GfcD	1:1	–34.64	–30.30	–14.57	28,350	1.0*	
**Biolayer interferometry**	**Model**	**Range (**μM)	***ka***_***1***_ **(M**^**–1**^** s**^**–1**^**)**	***kd***_***1***_ **(s**^**–1**^**)**	***K***_***D***_ **(nM)**	***ka***_***2***_ **(M**^**–1**^** s**^**–1**^**)**	***kd***_***2***_ **(s**^**–1**^**)**
GfcB-biotin, GfcC	1:1	0.2–1.6	52,268	0.0196	374	n.a.	n.a.
GfcB-biotin, GfcC	1:1 with Δ*conf* ^†^	0.2–1.6	59,934	0.0603	1,006	0.01105	0.0150
GfcC-biotin, GfcB	1:1	0.1–1.6	71,698	0.0126	175	n.a.	n.a.
GfcC-biotin, GfcB	1:1 with Δ*conf*	0.1–1.6	80,419	0.0328	407	0.00745	0.0107
GfcD-biotin, GfcB	1:1	3.75–30	652	0.00926	14,200	n.a.	n.a.
GfcD-biotin, GfcC	1:1	7.5–60	111	0.02858	256,800	n.a.	n.a.
GfcD-biotin, GfcBC	1:1	3.75–15	781	0.00716	9,163	n.a.	n.a.

*n set as constant since titration lacked inflection point.

^†^Fit to one-site binding model with subsequent conformation change of *ka*_2_ and *kd*_2_.

n.a. not applicable to model.

### GfcD binding to GfcB and GfcC

**Fig 5:**
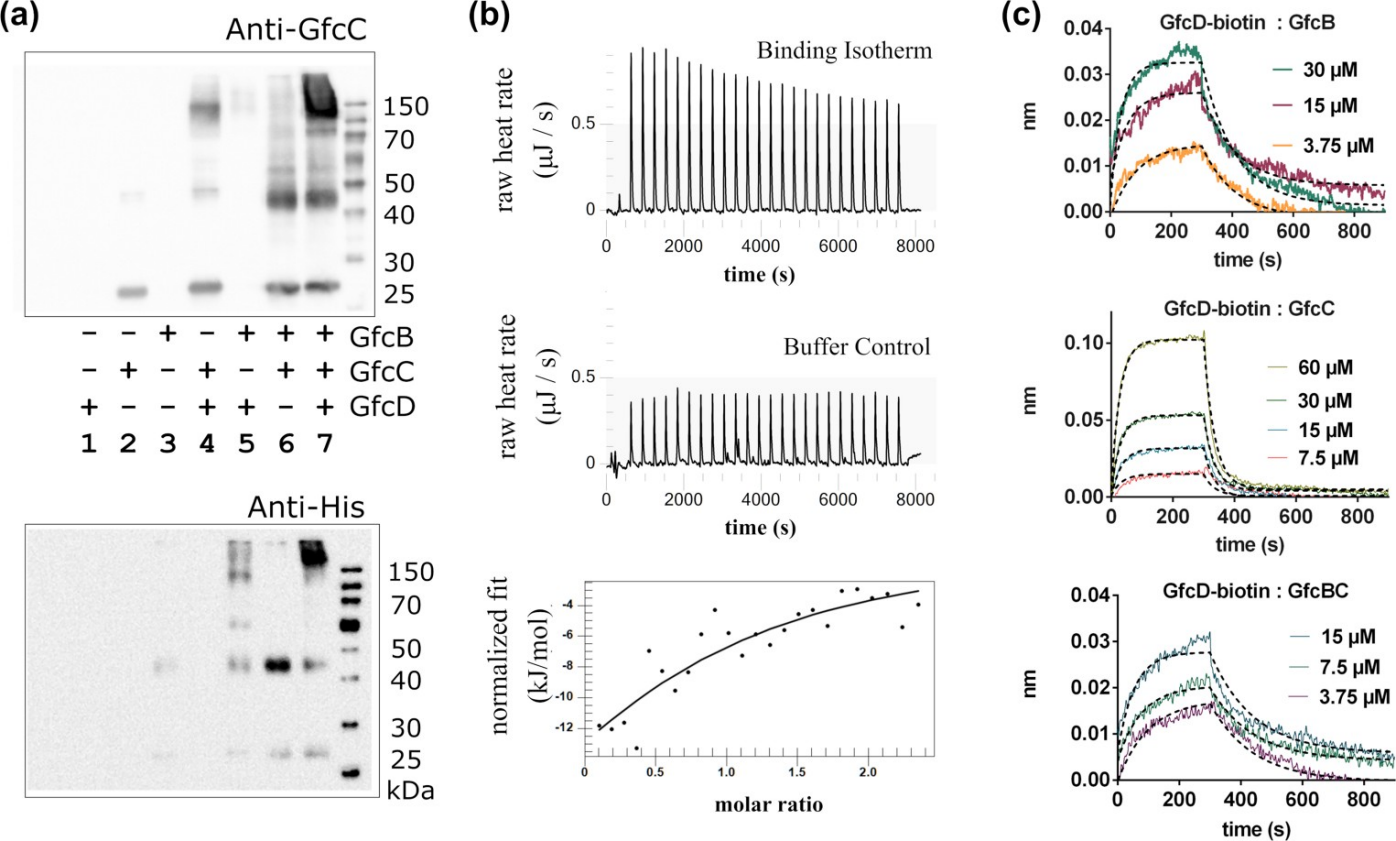
Interaction of GfcD with GfcB and GfcC. **(a)** Purified GfcB-6XHis, GfcC, and/or GfcD were cross-linked by DSP and run on SDS-PAGE. Western blotting identified 150 kDa complexes of GfcC-GfcD (**lane 4**), 50 kDa heterodimers with GfcB-GfcC (**lane 6**) and large complexes of GfcB, GfcC, and GfcD (**lane 7**). **(b)** Isothermal titration calorimetry of GfcBC into GfcD measured the heats of each injection (top) which were subtracted by heats of control injections of GfcBC into buffer (middle) and fit (bottom) to measure the affinity near 28 μM *K*_D_ for GfcBC. (**c**) BLI for the interactions of immobilized biotinylated GfcD with GfcB, GfcC, or the GfcBC complex at concentrations from 3.75–60 μM. Traces for GfcD-GfcB and GfcD-BC are a moving average over 20 data points to aid comparison against the fit.

To examine interactions between purified His6-GfcB and GfcC (expressed from pMCSG7) and GfcD (expressed from pMCSG26), we blotted with combinations of the proteins that had been cross-linked in solution at amine groups with dithiobis[succinimidyl propionate] (DSP). We saw no evidence for GfcC forming oligomers and observed a single band of expected 23 kDa size (Fig 5A, lane 2). The crystal structure of GfcB (PDB id 2IN5) showed a homodimer. Similarly, after crosslinking, His6-GfcB was observed as a 45–50 kDa band in both lanes 3 and 5 (Fig 5A**)** matching twice the expected size of His6-GfcB. After mixing and crosslinking, His6-GfcB and GfcC produced a heterodimer at 45–50 kDa as identified by anti-His and anti-GfcC blotting (Fig 5A, lane 6). His6-GfcB bound GfcD as detected by anti-His staining near the high MW of GfcD in lane 5 and, likewise, when GfcC cross-linked with GfcD similarly in lane 4. High molecular weight complexes in the range of 150–250 kDa were found when GfcB, GfcC, and GfcD were crosslinked together (Fig 5A, lane 7).

We then examined the GfcB-GfcC heterodimer and GfcD interactions by calorimetry (ITC) and biolayer interferometry (BLI). The necessity of concentrations greater than 1 mg mL^–1^ for GfcD made testing GfcD binding to the GfcB-GfcC heterodimer more difficult. With ITC, we found that GfcD had an enthalpically driven interaction with GfcB-GfcC (GfcBC) with *K*_D_ of 28 μM (Fig 5B and Table 2). This was a 300-fold lower affinity than the interaction of GfcB and GfcC. At the limits of protein concentration that we could test, we did not see a visible inflection point in the titration curve. The value for stoichiometry (*n*) was held at 1.0 to provide estimates of the enthalpically-driven binding of GfcBC with GfcD at Δ*H* of –30 kJ/mol, and a negative change in entropy upon binding. Heats of dilution of GfcBC were constant and of low magnitude, and titrating GfcBC into the volume of GfcD had measureably larger energy responses and demonstrated a titration-dependent response of a binding interaction. The response was limited by the conditions *c* = *K*_A_* [M] * *n*, (Eq 3 in [39]), where the constant *c* is below the ideal range 1 < *c* < 500 limited by available concentration of the protein. The accurate determination of the binding constant requires concentrations that satisfy the relationship of Eq 3, and binding isotherms with low c values have broad transitions as we saw with the GfcD that make identification of an equivalence point difficult. We were limited by the concentrations of GfcD possible to obtain for ITC studies and adjusted by comparing with a different biophysical method next.

**Fig 6:**
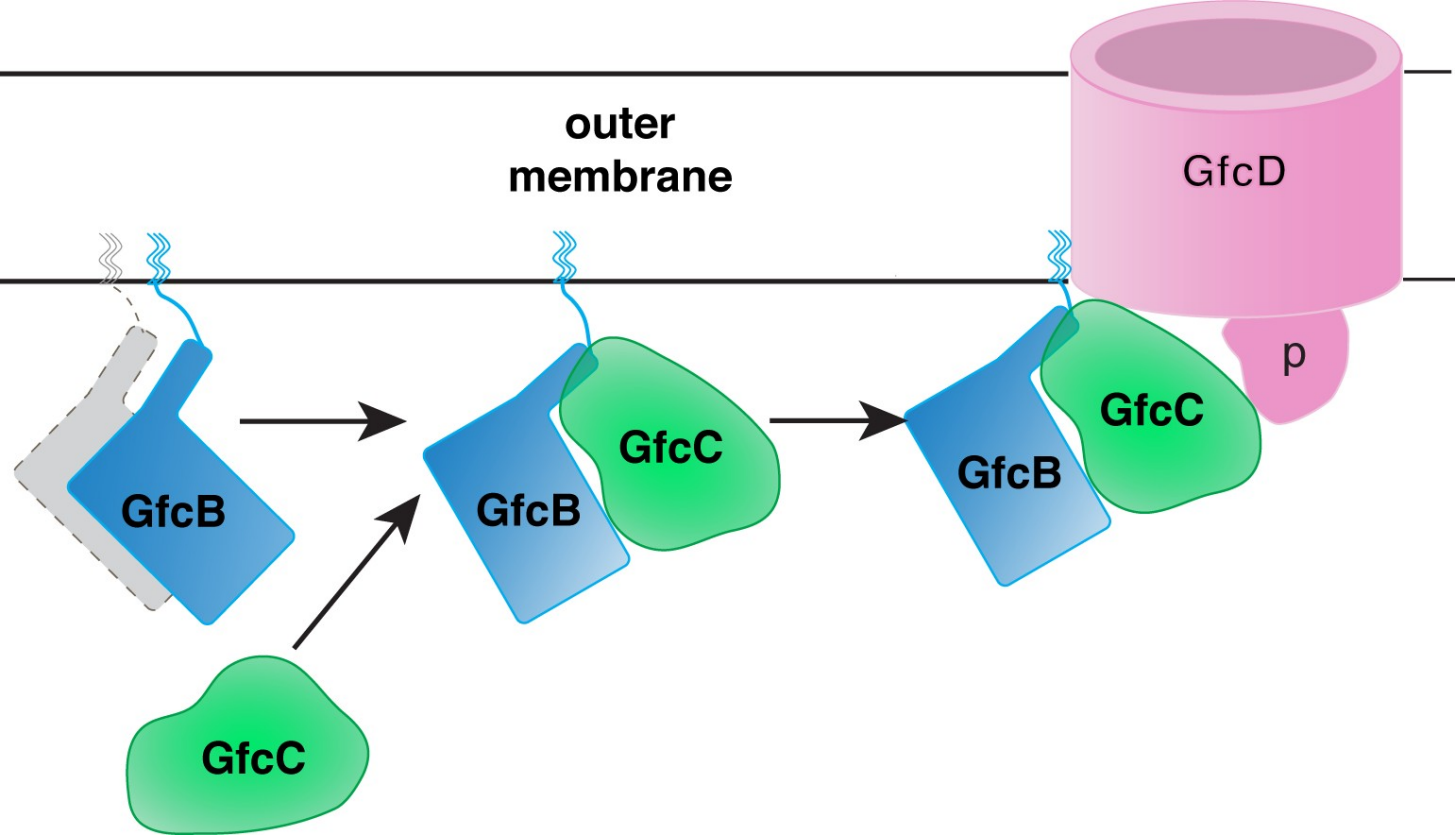
Possible scheme for the formation of the GfcB-GfcC heterodimer and complex with GfcD. The lipoprotein GfcB (possibly a dimer based on PDB entry 2IN5 and the crosslinking experiment in Fig 5A) is anchored to the inner leaflet of the outer membrane and forms a heterodimer with GfcC before binding to GfcD as suggested by the experiment shown in Fig 5A and the relative binding affinities to GfcD (Fig 5C). Domain labeled **p** is the predicted periplasmic domain of GfcD (see also S3 Fig) which may make interactions with GfcC and/or GfcB, and/or gate the opening of the GfcD barrel.

The estimated *K*_D_ were compared with the biolayer interferometry (BLI) assay. The BLI studies required only 10 μg mL^–1^ of biotinylated GfcD when anchored to the SA sensor. We measured binding (Fig 5C) between GfcD and GfcBC with a *K*_D_ of 9.1 μM, while GfcD and GfcB alone had a *K*_D_ of 14.2 μM and GfcC had a *K*_D_ of 256.8 μM. In comparison, the GfcBC binds with higher affinity to GfcD than either component alone. We hypothesize that the lipoprotein GfcB at the outer membrane binds to the periplasmic GfcC, then the complex colocalizes with GfcD forming a heteromeric complex as shown in Fig 6.

## Discussion

As one of seven proteins encoded by the *gfc* operon, GfcD is expected to have a function linked to capsular polysaccharide export. Other members of the operon have functions inferred from homology to the Group 1 capsule export proteins. GfcE is 95% similar to the Group 1 protein Wza, an octamer that spans the periplasm and forms an α-helical transmembrane pore for exopolysaccharide export. Wzb and Wzc, the tyrosine phosphatase and kinase, respectively, similarly serve as homology models for Etk and Etp. The remaining proteins have poorly established function. GfcD has been annotated as a member of the ‘domains of unknown function’ (DUF940) family including *E*. *coli* paralog YjbH, *Vibrio anguillarum* homolog WbfB, and related proteins of Shigella, Burkholderia, Vibrio, and other Gram-negative species. A recent study in the hypervirulent fish pathogen *Aeromonas hydrophila*, showed that GfcD was essential for virulence and biofilm formation, and induced an adaptive immune response in susceptible fish [25]. The overexpression and purification of the GfcD allowed our group to characterize where the protein localizes in *E*. *coli*, to measure binding interactions with other members of the *gfc* operon, and to measure secondary structure relating to the overall fold.

### GfcD is in the outer membrane

The overwhelming majority of the GfcD protein localized in the enteropathogenic E. coli outer membrane (Fig 3B). While there is widespread annotation of GfcD in online databases as a putative lipoprotein, and cysteine residues within the signal sequence are a potential site for acylation, the results presented above show that this assumption is incorrect. The lack of an acyl-group on the amino-terminus and the presence of a signal 1 peptidase site at amino acid E22 of GfcD makes it unlikely that it anchors in the membrane due to acylation at the amino terminus. Instead, GfcD is predicted to insert into the outer membrane as an integral membrane protein.

### Domain structure for GfcD

Circular dichroism spectroscopy for GfcD match a secondary structure like the canonical outer membrane protein OmpW. Topology predictions of 26-strands in a β-barrel domain of GfcD may provide a large channel for the export of O-antigen polysaccharides or other substrates like the exopolysaccharide system of AlgE, a bacterial portal for alginate [40]. We also found a higher proportion of α-helical structure in GfcD than in β-barrel structures such as OmpW, and our sequence-based topology model suggests a central region of the protein may be composed of an α/β domain that could act as a periplasmic “plug”, or a scaffold for protein-protein interactions. Such a plug domain might resemble TonB-dependent OM receptors (FhuA, FecA, FepA, and BtuB) that have periplasmic domains that can act as plugs or corks that occlude the interior of the β-barrel, but actively bind and translocate a siderophore across the channel with energy provided by TonB [41]. Similarly, the lipopolysaccharide assembly protein LptD forms a large 26-strand β-barrel in complex with LptE, which acts as a plug in the complex structure [42, 43]. The SEC-MALS analysis of the GfcD:DDM protein-detergent complex revealed that it was a 76 kDa monomeric protein that had 158 kDa in detergent mass which is twice the expected mass of an empty DDM micelle.

### A large barrel or a multi-barrel protein?

Juxtaposed against these models of plug and barrel architectures, a recent analysis of bacterial structure predictions based on contact-maps suggest that many outer membrane protein families may actually be comprised of multiple barrels [44]. The authors proposed that the YjbH/GfcD family of proteins may adapt a two-barrel structure comprised of a 12-stranded β-barrel of the FapF family, a middle globular binding protein, and an unknown 12-stranded β-barrel [44]. Although intriguing, there have been no structures determined of this type of dual barrel membrane protein to the authors’ knowledge. Nevertheless, the GfcD dual barrel structure was recently predicted with high confidence by AlphaFold (https://alphafold.ebi.ac.uk/entry/P75882) [45]. This model has α 12-stranded barrel, followed by an aβ-domain with 5 β-strands and 3 α-helices, followed by a 13-stranded β-barrel (S3 Fig). Our observations of the limited proteolysis of GfcD forming two stable 30-kDa fragments in LDAO and other smaller micelle detergents may provide support for such multi-barrel structures within GfcD.

### GfcD function may require binding of GfcBC

GfcD may function as an export portal similar to other exopolysaccharide systems such as AlgE [46] that utilize an outer membrane β-barrel. Involvement of the homologous operons *E*. *coli yjbEFGH* [26] and *V*. *anguillarum orf1-wbfDCB* [24] in exopolysaccharide production would seem to support such a role. Operons like *yjbEFGH* may not require *wza-wzb-wzc* homologs encoded nearby or, when present, these genes may be transcribed in a different direction as is the case with the *orf1-wbfDCB* operon. There are 1–4 base pair overlaps between both the *gfcB-gfcC* and *gfcC-gfcD* gene pairs which is also seen in homologous operons. Short tandem overlapping sequences may enable translational coupling to preserve stoichiometries of the *gfc* proteins [47]. Another homolog, the 995-amino-acid OtnG protein of *Burkholderia thailandensis*, has 26.8% identity to GfcC at the N-terminus and 33.8% identity to GfcD at the C-terminus, resembling a fusion of GfcC-GfcD. The gene overlaps and fusion homologs suggest coupled functions of these gene products.

We identified interactions between GfcB, GfcC, and GfcD in EPEC. GfcB-His6 behaved predominantly as a dimer when expressed by itself (Fig 5A, lower panel, lane 3), consistent with the dimer observed in the crystal structure of GfcB (YmcC) (PDB id 2IN5). GfcB or GfcC each bound to GfcD with a high μM affinity. Mixed solutions of GfcB and GfcC formed a high-affinity heterodimer (Fig 4); the heterodimer was also observed in the *in vivo* crosslinking experiment (Fig 5A, lane 6). This GfcB-GfcC heterodimer interacts with GfcD at higher affinity than either of the components alone (Fig 5). In vivo, the GfcB-GfcC heterodimer would be localized to the outer membrane because GfcB is a lipoprotein, effectively increasing its local concentration for interaction with GfcD. Protein-protein interactions with K_D_ values within the μM range are often transient, with lifetimes lasting in seconds, and yet play important functional roles within the cell including proteins that act as regulators of other cellular processes [48]. The protein-protein interactions identified in the GfcBCD proteins (shown schematically in Fig 6) complement previously characterized interactions between other *gfc* proteins. These include interactions between Etk and partner proteins GfcE, Etp, and GfcD that were identified by the large-scale tagging of engineered *E*. *coli* proteins and mass spectrometry [49].

In the AlphaFold model of the GfcD dual barrel structure (S3 Fig), the periplasmic middle domain of GfcD is positioned at the periplasmic opening of the second barrel. It is intriguing to speculate that the GfcB-GfcC heterodimer binds to this domain and regulates the function of GfcD (see Fig 6).

Unfortunately, we do not have direct data to annotate the function of GfcD. We presume that GfcE, the paralog of Wza, is the likely export portal for group 4 polysaccharide. GfcD, together with GfcB and GfcC may be an alternate export route for the polysaccharide, or perhaps a polysaccharide conjugated to a protein or lipid. It is noteworthy that *gfcA* is predicted to encode a small inner membrane protein rich in Ser and Thr residues that might be modified by O-antigens and then directed to GfcD for export.

GfcB/GfcC could play an accessory role in the export process. A search with the Dali server showed that GfcB has a similar fold to the LolA lipoprotein chaperone [33]. In the periplasm, LolA acts with LolB to transfer lipoproteins to the outer membrane. GfcBC may similarly shuttle ligands or aid the function of the outer membrane GfcD. GfcD may even have a similar role to the LptD lipopolysaccharide assembly protein to aid the insertion of LPS-like O-antigen polysaccharides into the outer membrane of EPEC. This system could operate in conjunction with the other export proteins of GfcE, Etk, and Etp or independently. The further structural study of the GfcBC complex, GfcD, and comparisons to known systems such as the Group 1 capsule secretion system and the LPS secretion systems may help to pin a function on the GfcBCD complex and its many orthologs from other species.

## Conclusion

Enteropathogenic *E*. *coli* GfcD is an integral outer membrane protein of a β-barrel fold with an internal globular domain. We have now identified the high-affinity interactions between components GfcB and GfcC, and their lower affinity binding to GfcD. The processes involved in the production of the group 4 O-antigen capsule remain unclear. The structural study of this protein complex could provide a new target for understanding polysaccharide assembly machinery at the outer membrane. Recent techniques for the structural and functional characterizations of large protein complexes including structure prediction and cryo-electron microscopy could be applied to better understand the outer membrane complex and its role in the O-antigen capsule production, export, and regulation.

## Experimental procedures

*E*. *coli* strains and plasmids, and DNA oligonucleotides are described in the, S1 and S2 Tables.

### Construct design and cloning

EPEC compatible vectors were generated as follows: The *gfcD* gene was cloned by PCR amplification of *E*. *coli* O127:H6 str. E2348/69 chromosomal DNA with primers 1 and 2 (S2 Table), followed by restriction digest (MfeI, PstI), and ligation (T7 ligase) into the previously digested pSA10 vector (S1 Table). pSA10 has a Tac promoter that constitutively expressed the gene at low levels but was also inducible with IPTG. The C-terminal His6-tag was modified in pSA10(*gfcD*) by QuikChange (Agilent) mutagenesis to expand the affinity tag to 10 histidine residues (primers 3 and 4). The contiguous *gfcBCD* fragment was similarly cloned into pSA10 (primers 5 and 6), and a His6-tag was introduced at the C-terminus of the *gfcB* by mutagenesis before the last codon (primers 7 and 8) to leave the overlapping bases between *gfcB-gfcC* intact.

For larger scale over-expression, we created T7-inducible expression plasmids (S1 Table). The *gfcD* gene was amplified by PCR (primers 15–18) and inserted into pMCSG26 and pBH31 vectors by ligation independent cloning (LIC) [50]. The pMCSG26 vector added a C-terminal polyhistidine tag to the polypeptide and kept the original amino-terminal signal sequence of GfcD. Alternatively, the pBH31 vector replaced the signal sequence with the *pelB* periplasmic export sequence, 6X-polyhistidine tag, and a TEV protease cleavage site. As above, the 10X His-tag was introduced into pBH31(*gfcB*) by mutagenesis (primers 19 and 20). The pET-Blue2(*gfcB*) construct was created by PCR amplification of the *gfcB* gene fragment (primers 9 and 10), digestion with NcoI and XhoI, and ligation into pETBlue2 (Novagen). The *gfcB* and *gfcC* genes were amplified by PCR (primers 11–14) and inserted into pMCSG7 by LIC. The pMCSG7 plasmid provided an amino-terminal 6X polyhistidine tag followed by a TEV cleavage site.

### Large scale expression of GfcD

The expression plasmids were transformed into BL21(DE3), C41(DE3), or Tuner^TM^(DE3)/pLacI competent cells. Forty mL of Terrific Broth (TB) containing 100 μg ml^–1^ ampicillin and 34 μg ml^–1^ chloramphenicol in a 250 mL flask was inoculated with a single colony. This culture was grown for 6–8 hours at 37°C, then used to inoculate four 1 L cultures of TB/amp media. When the optical density at 600 nm (OD600) of the culture exceeded 2.0, the temperature was lowered to 22°C and cells were induced with 0.05 mM IPTG for 8–12 hours. Cells were pelleted by centrifugation at 5000 *g* for 20 minutes at 4°C, re-suspended in 50 mM sodium phosphate, 20% w/v sucrose at pH 7.4, 10 μg mL^–1^ DNase, 0.1 mg mL^–1^ lysozyme, rocked for 10 minutes, and lysed by a single passage through a microfluidizer cooled at 4°C. The lysate was separated by centrifugation at 10,000 rpm for 20 minutes to clear cell debris, and the supernatant ultracentrifuged at 250,000 *g* in a Ti-70 rotor to sediment the membranes.

### Isolation and purification of GfcD

GfcD was extracted from the membranes in the presence of 5% Elugent^TM^ in 50 mM Tris-HCl, 150 mM NaCl at pH 7.4. Detergent-solubilized proteins were cleared by centrifugation at 20,000 *g* then filtered. We found that a 6X polyhistidine-tag was insufficient to bind to the resin. After addition of a 10X polyhistidine-tags to either the C- or N-termini, GfcD proteins bound the metal affinity resin of the HisPur (Thermo Fisher) cobalt affinity column and eluted in a gradient from 0–500 mM imidazole. The amino-terminal polyhistidine tags were cleaved overnight by addition of TEV protease at a 1:50 molar ratio (TEV:protein) while dialyzing against 20 mM Tris-HCl pH 8, and 1 mM DTT. Cleavage was confirmed by anti-His blotting and increased mobility on SDS-PAGE gels. GfcD was then bound to a SourceQ anion exchange column and eluted with a gradient of 0–1 M NaCl. Fractions containing GfcD were pooled and concentrated to 250 μL and exchanged into different detergents by passing over a Superdex 200 10/300 GL size exclusion column. GfcD was exchanged into buffers containing 0.06% w/v *n*-dodecyl-N,N-dimethylamine-N-oxide (LDAO), 1% n-octyl-β-D-glucopyranoside (OG), 0.2% *n*-nonyl-β-D-glucopyranoside (NG), 0.2% w/v *n*-decyl-β-D-glucopyranoside (DG), or 0.02% w/v *n*-dodecyl-β-D-maltopyranoside (DDM). Detergents were purchased from Anatrace and were of Anagrade quality. The protein was concentrated to >10 mg mL^–1^ as assessed by UV absorption.

### Expression and purification of GfcB and GfcC

GfcB and GfcC were expressed and purified similarly to GfcD described above, but as soluble proteins. Plasmids containing *gfcB* or *gfcC* without signal sequences were transformed into Tuner(DE3)/pLacI. A single colony was picked to inoculate a 20 mL TB culture that was grown overnight and subsequently inoculated into 1 liter TB cultures and grown at 37°C. For pMCSG7(*gfcC*), the culture was induced when OD600 reached 0.6, and induction continued overnight at 22°C. For pMCSG7(*gfcB*) or pETBlue2(*gfcB*), induction started after OD600 reached 2.0 and continued at 30°C for 4 hours. Cells were collected by centrifugation at 5,000 *g* for 20 minutes at 4°C and then re-suspended in 100 mL of 50 mM Tris-HCl, 300 mM NaCl, and 5 mM imidazole at pH 7.5. The suspension was lysed by sonication in a Branson sonifier for 5 min while cooled on ice. Lysates were clarified by centrifugation at 20,000 rpm in a JA-20 rotor (Beckman) for 20 minutes at 4°C, filtered, and passed over a HisPur cobalt resin. Fractions containing GfcB or GfcC were pooled, and His6-tags were removed by incubation with TEV protease. Uncleaved proteins and protease were removed by repeating the affinity column and collecting the flow-thru, and fractions containing the untagged proteins were pooled. Results of the GfcB and GfcC purifications are shown in S1 File.

### Preparation of anti-GfcC and anti-GfcD antibodies

Polyclonal antibodies were raised against GfcD purified from pSA10(*gfcD*) (Covance) or GfcC expressed from pMCSG7(*gfcC*) (Thermo Scientific). The 77-day protocol involved three applications of the purified antigen and serum collection from the rabbits at 31, 52, and 77 days. Serum from the 2^nd^ and 3^rd^ collections were used without further purification. These polyclonal and additionally a monoclonal anti-polyhistidine tag antibodies (clone His-1, Sigma) identified target proteins in western blots.

### Identification of N-terminus

Recombinant GfcD with native signal sequence was purified from BL21(DE3) / pMCSG26(*gfcD*). GfcD was loaded on a 10% SDS-PAGE gel without heat denaturing the protein samples. The GfcD band was excised from the gel slice, digested with trypsin and chymotrypsin, and fragments were identified by LC-MS/MS at the Proteomics & Peptide Synthesis Core (University of Michigan). For amino-terminal sequencing, GfcD was transferred to PVDF membranes, stained with GelCode blue, and the band excised for Edman degradation sequencing at the same core facility. N-terminal amino acid sequencing of GfcD bound to a PVDF membrane was performed by automated Edman sequencing in an Applied Biosystems 494HT sequencer equipped with a 610A data analysis module (version 2.1). Data interpretation and review were performed at the core facility.

### Multi-angle light scattering (MALS)

GfcD purified from the pBH31 construct was diluted to 2 mg/mL in a buffer of 10 mM Tris-HCl pH 8, 100 mM NaCl, 1 mM TCEP, 1 mM EDTA, and 0.02% w/v DDM. Size exclusion chromatography combined with multi-angle light scattering measurements were made with the GE Healthcare AktaMicro system connected to a MALS scattering detector and a differential refractometer (Wyatt Dawn Heleos^®^ II and Optilab^®^ T-rEX^™^, respectively). Theoretical specific refractive index increment (*dn*/*dc*) values [34] of 0.185 ml g^–1^ and 0.133 ml g^–1^ were used for the protein and DDM detergent, respectively. A theoretical extinction coefficient of 1.717 for 1 mg mL^–1^ concentration was used for GfcD. Fifty μL of protein was applied to a WTC-030S5 SEC silica column and eluted with 14 mL of buffer at a flow rate of 0.4 mL per minute. The data were recorded and processed with the ASTRA 6 software (Wyatt Technologies) and are shown in S2 Fig. The output of the protein conjugate analysis is shown in S3 Table.

### Circular dichroism spectroscopy

GfcD was exchanged into 50 mM sodium phosphate and 0.02% DDM then diluted to 0.086 mg mL^–1^ concentration. Protein or buffer alone were added to a 1 mm cuvette, and 10 spectra from 185–260 nm were collected on a Jasco 715 CD spectrometer. Spectra were converted from millidegrees to molar ellipticity units, and deconvoluted with SOMCD [37] to obtain secondary structures. The OmpW spectra was obtained from the Protein Circular Dichroism Data Bank [51].

### Sucrose gradient fractionation

EPEC *gfcD*::*kan* / pSA10(GfcD) was grown overnight in 5 mL of LB broth containing 100 μg mL^–1^ ampicillin. The following day, 100 mL of LB with 100 μg mL^–1^ ampicillin was inoculated with 1 mL of overnight culture and grown until OD600 reached 0.6. IPTG (0.5 mM) was added to the cultures and kept at 30°C for an additional 7 hours with shaking. Lysis and harvesting of membranes were described above in *Large scale expression of GfcD*. Membrane pellets were re-suspended in 50 mM sodium phosphate pH 7.4, 10% glycerol, 20% sucrose, and 250 μL of the membrane solution was added to the top of a discontinuous gradient of layered solutions—4 mL of 70% sucrose, 4 mL of 60% sucrose, and 3.75 mL of 20% sucrose—in an ultraclear tube. The tubes were loaded in a Beckman SW-41 swinging bucket rotor and centrifuged at 28,000 rpm (134,000 *g*) for 16 hours at 4°C. At the end of the sedimentation, tubes were punctured at the bottom with a hot needle and 800 μL fractions were collected. Fractions were analyzed for NADH oxidase activity, run on SDS-PAGE and then blotted.

### EPEC growth curves

EPEC/pSA10, EPEC/pSA10(*gfcD-His6*), EPEC/pSA10(*gfcC-His6*), and EPEC/pSA10(*gfcB-His6-CD*) were cultured overnight in LB-Amp at 37°C. On the next day, 100 μL of LB/amp containing 100 μM IPTG were inoculated with 10 μL of a 1/10 diluted overnight culture. The OD600 for triplicate wells was measured each hour.

### Crosslinking and pull-down experiment

To promote entry of crosslinking reagents into the cells, we grew an EPEC *etk*::*kan* mutant, which lacks a capsule, transformed with pSA10(*gfcBCD*) or pSA10(*gfcB-His6-CD*). After growth in 100 mL LB cultures at 37°C and induction with 0.1 mM IPTG for 1 hour, cell pellets were collected by centrifugation at 5000 *g* for 10 minutes and washed by re-suspension in 10 mL PBS and repeated 3 times to remove free amines. Crosslinking was started by incubating the cells in PBS containing 5 mM dithiobis[succinimidyl propionate] (DSP, from Pierce) at 4°C while rocking. Cells were lysed by sonication, pelleted, and the supernatant was transferred into 1.5 mL tubes then spun in a Fiberlite F50L rotor (Thermo Scientific) at 95,000-g for 45 minutes at 4°C to obtain the membrane fraction. Membranes were solubilized as described above and 200 μL was mixed with 60 μL of HisPur NiNTA resin (Thermo Scientific) for 30 minutes. The resin was washed 4 times with 250 μL of buffer A (50 mM sodium phosphate, 150 mM NaCl, 0.1% DDM pH 7.4) and proteins were eluted with 50 mM EDTA. Crosslinked samples were mixed with 5X sample buffer without DTT and boiled for 6 minutes, then loaded onto 13% SDS-PAGE gels. Proteins were transferred to blots and blotted with anti-polyhistidine, anti-GfcC, or anti-GfcD antibodies.

### Isothermal titration calorimetry

All titrations were performed using a NanoITC calorimeter (TA instruments) with a buffer of 20 mM Tris-HCl pH 7.5, 150 mM NaCl and using a 250 μL syringe. GfcB (2.5 mg mL^–1^) and 0.6 mg mL^–1^ GfcC were separately dialyzed overnight against the buffer. Titrations were initiated with a 2 μL injection (later removed from analysis), followed by 24 x 10 μL injections at 300 second intervals. All titrations were completed at 25°C while stirring at 300 rpm. The heats of dilution were measured by titrating 100 μM GfcB or 160 μM GfcB-GfcC into buffer. Experimental titrations of 100 μM GfcB were injected into 10 μM GfcC in 20 mM Tris-HCl pH 7.5, 150 mM NaCl. Similarly, 160 μM of the GfcB-GfcC complex were injected into 20 μM GfcD in 20 mM Tris-HCl pH 7.5, 150 mM NaCl, 0.02% DDM. Data were fit in NanoAnalyze 2.4.1 (TA Instruments), and the area under each injection was corrected by subtracting the matching area under the buffer blank control prior to fitting.

### Biolayer interferometry

After removal of polyhistidine tags, the GfcB (pMCSG7), GfcC (pMCSG7) and GfcD (pBH31) proteins were diluted to 1 mg/mL and exchanged into 0.02% DDM in PBS. The proteins were labeled with Biotin-PEG4-NH_2_ and excess label was removed by dialysis. Presence of biotin tag was confirmed by western blotting and detection with streptavidin-HRP. Interactions of the GfcB, GfcC, and GfcD proteins were measured by BLI in an OctetRed system (ForteBio). Streptavidin sensor tips (SA) were incubated for 5 minutes with 10 μg mL^–1^ of labeled proteins to reach saturation. Kinetic assays were conducted at 25°C with an interaction buffer of 20 mM Tris-HCl pH 7.5, 150 mM NaCl, and 0.1 mg mL^–1^ BSA (to minimize non-specific interactions). For interaction studies involving GfcD, the buffer was augmented with 0.02% DDM detergent. The baseline response was measured over 60 seconds, then sensors were moved into a 200 μL solution of the protein analyte solution at concentrations of 0.1 nM to 60 μM. The association was measured over 600 seconds, then the tip was moved into buffer solution for 1200 seconds to measure the dissociation. Data were initially processed in Data Analysis 7.0.0.34 (ForteBio) to subtract the experimental sensor data by parallel reference sensors of an unlabeled control for non-specific signals. Afterwards, data were exported for fitting with a one-site association-dissociation model in PRISM 6.02 (GraphPad Software). The interactions of GfcB (expressed from pMCSG7 or pETBlue2) with GfcC were fit by the one-site binding followed by conformation change model^23^. In this model, GfcB and GfcC bind to form a complex AB that undergoes a conformational change to AB*. This model is described by Eqs 1–3 below.


$$d[B]/dt=-ka_{1}\;[A][B]+kd_{1}\;[AB]$$ (1)

$$d[AB]/dt=ka_{1}\;[A][B]-kd_{1}\;[AB]-ka_{2}\;[AB]+kd_{2}\;[AB*]$$ (2)

$$d[AB*]/dt=ka_{2}\;[AB]-kd_{2}\;[AB*]$$ (3)

Fitting of the conformational change model was implemented in OCTAVE [52] by numerical minimization of the sum of squared errors in predicted fits (SSE) to experimental binding responses with the parameters (*ka*_1_, *kd*_1_, *ka*_2_, *kd*_2_) fitted globally.

### In vitro crosslinking

Purified His6-GfcB (from pMCSG7), GfcC (pMCSG7), and GfcD (pBH31) were separately dialyzed overnight against 20 mM Hepes, 150 mM NaCl, and 0.06% DDM at pH 7.5. The proteins were mixed at 5 μM final concentrations in 30 μL volumes. After equilibration for 10 minutes at room temperature, 1 μL of 10 mM DSP was added to crosslink interacting proteins. After 1 hour at room temperature, 5 μL of 200 mM Tris-HCl pH 7.5 were added to inactivate the crosslinker reagent. Proteins were separated on SDS-PAGE, transferred to blots, and blotted with anti-polyhistidine and anti-GfcC antibodies.

## Supporting information

S1 Table Bacterial strains and plasmids used in the research. (PDF)

S2 Table Oligonucleotide (primer) sequences for cloning and mutagenesis. (PDF)

S3 Table Protein conjugate analysis of SEC-MALS result for GfcD:DDM shown in S2 Fig. (PDF)

S1 Fig Peptides identified from MS/MS of purified GfcD expressed in EPEC. The first peptide sequence identified EVLTYP… by MS/MS was identical to that determined from N-terminal sequencing.
(TIF)

S2 Fig GfcD in DDM detergent behaves as a monomer by size exclusion chromatography with multi-angle light scattering (MALS). **(a)** The red trace is the light scattering shown on the left Y-axis. The numbers identified in the legend indicate molecular masses (right Y-axis) calculated by protein conjugate analysis of the MALS data for peak 1 (S3 Table). Fifty μL of protein was applied to a WTC-030S5 SEC silica column and eluted with 14 mL of buffer at a flow rate of 0.4 mL per minute. Other details in **Experimental procedures**. **(b)** Similar representation showing light scattering (red), differential refractive index (blue), and protein absorption curves (dashed green). Protein molar masses are shown in black. Only the first peak contains a protein mass consistent with GfcD. The second peak is primarily DDM micelles.
(TIF)

S3 Fig Tertiary structure of GfcD predicted by AlphaFold (entry P75882) [45], shown is the double-barrel structure with a predicted periplasmic globular domain (labeled P). (TIF)

S1 File GfcB and GfcC protein purifications; GfcD SEC profiles in detergents. (PDF)

S1 Raw images Blot details and original scans for Figs 3B, 3C, 3E and 5A. (PDF)
