# Supplementary material for: Escherichia coli O127 group 4 capsule proteins assemble at the outer membrane
Source: PLoS One. 2021 Nov 15;16(11):e0259900. doi: 10.1371/journal.pone.0259900 (PMC8592465; doi:10.1371/journal.pone.0259900)
Supplement: S1 Table — (PDF) [file pone.0259900.s001.pdf]

S1 Table. Bacterial strains and plasmids.

	Strain or plasmid	Description of plasmid	Source§ of strain or plasmid
	Strain		
**1**	E2348/69 EPEC	Enteropathogenic *E. coli* O127:H6	Lab stock
**2**	EPEC *gfcD::kan*	*gfcD* gene disruption	[1]
**3**	EPEC *etk*::*kan*	*etk* gene disruption	[1]
	Plasmid		
**1**	pSA10	P*tac,* Amp^R, encodes C-terminal His_6	
**2**	pSA10(*gfcC*)	GfcC(1–248)-His_6	[1]
**3**	pSA10(*gfcD*)*	GfcD(1–698)-His_6 / primers 1 & 2†	This work.
**4**	pSA10(*gfcD-10XHis*)*	GfcD(1–698)-His_{10} / primers 3 & 4	Derived from #3
**5**	pSA10(*gfcBCD*)*	GfcB, GfcC, GfcD / primers 5 & 6	This work.
**6**	pSA10(*gfcB*-His-*CD*)*	GfcB(1–213)-His_6, GfcC, GfcD / Primers 7 & 8	Derived from #5
**7**	pETBlue2	P_{T7}, Amp^R encodes C-terminal His_6	Novagen
**8**	pETBlue2(*gfcB*)	GfcB(18–214) / primers 9 & 10	This work.
**9**	pMCSG7	P_{T7}, Amp^R, N-terminal His6-TEV_site-(LIC‡)	[2]
**10**	pMCSG7(*gfcB*)*	GfcB(17–214) / primers 11 & 12	This work.
**11**	pMCSG7(*gfcC*)*	GfcC(22-248) / primers 13 & 14	This work.
**12**	pMCSG26	P_{T7}, encodes (LIC)-C-terminal His6 or His10	[3]
**13**	pMCSG26(*gfcD*)*	GfcD(1–698)-His_{10}, primers 15 & 16	This work.
**14**	pBH31	P_{T7}, pelB_signal-His6-TEV_site-(LIC)	
**15**	pBH31(*gfcD*)*	His_6-GfcD(22–698) / primers 17 & 18	This work.
**16**	pBH31(*gfcD*-10xHis)*	His_{10}-GfcD(22–698) / primers 19 & 20	Mutagenesis introduced 10X His-tag from #15

* These plasmids are available from AddGene, https://www.addgene.org/Mark_Saper/, accession IDs 176191–176194 and 176196–176200.

† Primers are listed in Table S2.

‡ LIC: Ligation independent cloning.

§ Bracketed items are reference citations.

References cited:

1. Peleg A, Shifrin Y, Ilan O, Nadler-Yona C, Nov S, Koby S, et al. Identification of an Escherichia coli operon required for formation of the O-antigen capsule. J Bacteriol. 2005;187(15): 5259–5266. doi: 10.1128/JB.187.15.5259-5266.2005
2. Stols L, Gu M, Dieckman L, Raffen R, Collart FR, Donnelly MI. A new vector for high-throughput, ligation-independent cloning encoding a tobacco etch virus protease cleavage site. Protein Expr Purif. 2002;25(1): 8–15. doi: 10.1006/prep.2001.1603
3. Eschenfeldt WH, Lucy S, Millard CS, Joachimiak A, Mark ID. A family of LIC vectors for high-throughput cloning and purification of proteins. In: Doyle SA, editor. High Throughput Protein Expression and Purification. Humana Press; 2009. pp. 105–115. doi: 10.1007/978-1-59745-196-3
