# Supplementary material for: Escherichia coli O127 group 4 capsule proteins assemble at the outer membrane
Source: PLoS One. 2021 Nov 15;16(11):e0259900. doi: 10.1371/journal.pone.0259900 (PMC8592465; doi:10.1371/journal.pone.0259900)
Supplement: S2 Table — (PDF) [file pone.0259900.s002.pdf]

S2 Table. Oligonucleotides for cloning and mutagenesis.

	Primer	Sequence (5´→3´)*	Use
**1**	pSA10_gfcD_F	CATTAGCAATTGATGAAGAAGAACTCTTATCT	Cloning (MfeI)
**2**	pSA10_gfcD_R	ATGCCTGCAGTTAGTGATGGTGATGGTGATGTTGTCCAACCGGTACTTC	Cloning (PstI)
**3**	pSA10_gfcD_his_10X_F	GTTGGACAACATCACCATCACCATCATCACCACCATCACTAACTGCAG	Mutagenesis
**4**	pSA10_gfcD_his_10X_R	CTGCAGTTAGTGATGGTGGTGATGATGGTGATGGTGATGTTGTCCAAC	Mutagenesis
**5**	pSA10_gfcBCD_F	TATAACCCGGGATGCGCCCTCTTATTTTATCGAT	*gfcBCD* XmaI
**6**	pSA10_gfcBCD_R	TTATAGTCGACTCATTGTCCAACCGGTACTTCTCGCTC	*gfcBCD* SalI
**7**	pSA10_gfcB-his-CD-F	GACGTTTCTTAAACCCGCACATCACCATCATCACCACCCATGAATAAATTACAG	Mutagenesis
**8**	pSA10_gfcB-his-CD-R	CTGTAATTTATTCATGGGTGGTGATGATGGTGATGTGCGGGTTTAAGAAACGTC	Mutagenesis
**9**	pETBlue2-gfcB_F	CTAGACCCATGGCGCACAGCCAGCAAAGTATG	Cloning (NcoI)
**10**	pETBlue2-gfcB_R	CGTCTCGAGTGGTGCGGGTTTAAGAAACGT	Cloning (XhoI)
**11**	pMCSG7_gfcB_F	**TACTTCCAATCCAAT**GCTACGCACAGCCAGCAAAGTATG	LIC cloning
**12**	pMCSG7_gfcB_R	**TTATCCACTTCCAATG**TCATGGTGCGGGTTTAAGAAAC	LIC cloning
**13**	pMCSG7_gfcC_F	**TACTTCCAATCCAAT**GCTGCGCAAGGAATGGTGACT	LIC cloning
**14**	pMCSG7_gfcC_R	**TTATCCACTTCCAATG**TCACTCAGGAACACGTTGCG	LIC cloning
**15**	pMCSG26_gfcD_F	**GTCTCTCCCA**TGAAGAAGAACTCTTATCTTTTAAGC	LIC cloning
**16**	pMCSG26_gfcD_R	**TGGTGGTGCCCA**TGGTGATGGTGGGCACTTTGTCCAACCGGTACTTCTCG	LIC cloning
**17**	pBH31_gfcD_F	**TACTTCCAATCCAAT**GCCGAAGTATTAACCTACCCGGA	LIC7
**18**	pBH31_gfcD_R	**TTATCCACTTCCAATG**TTATTATTGTCCAACCGGTACTTC	LIC7
**19**	pBH31_gfcD_his_10X_F	CGATGGCCCACCATCATCACCACCACCACCATCATCATTCTTCTGG	Mutagenesis
**20**	pBH31_gfcD_his_10X_R	CCAGAAGAATGATGATGGTGGTGGTGGTGATGATGGTGGGCCATCG	Mutagenesis

*Notes: Underlined sequences are restriction enzyme sites. **Bold** sequences are ligation-independent cloning sites.
