# Supplementary material for: Escherichia coli O127 group 4 capsule proteins assemble at the outer membrane
Source: PLoS One. 2021 Nov 15;16(11):e0259900. doi: 10.1371/journal.pone.0259900 (PMC8592465; doi:10.1371/journal.pone.0259900)
Supplement: S3 Table — (PDF) [file pone.0259900.s003.pdf]

S3 Table. Protein Conjugate Analysis of SEC-MALS result for GfcD:DDM shown in S2 Fig.

Peak Results	Peak 1	Peak 2
**Masses**		
**Calculated Mass (μg)**	62.98	130.95
**Protein mass (μg)**	2.0573×10^{1}	6.1594×10^{-1}
**Modifier mass (μg)**	4.2409×10^{1}	1.3034×10^{2}
**Molar mass moments (g/mol)**		
**Mn**	2.343×10^{5} (±1.653%)	7.606×10^{4} (±1.801%)
**Mw**	2.344×10^{5} (±1.650%)	7.606×10^{4} (±1.801%)
**Polydispersity**		
**Mw/Mn**	1.001 (±2.336%)	1.000 (±2.548%)
**Mw/Mn (Protein)**	1.001 (±2.336%)	1.031 (±2.758%)
**Mw/Mn (Modifier)**	1.000 (±4.183%)	1.000 (±3.763%)
**rms radius moments (nm)**		
**Rz**	3.4 (±460.4%)	5.1 (±215.7%)
**Protein molar mass moments (g/mol)**		
**Mn (protein)**	7.654×10^{4} (±1.653%)	3.480×10^{2} (±1.963%)
**Mw (protein)**	7.662×10^{4} (±1.651%)	3.586×10^{2} (±1.938%)
**Modifier molar mass moments (g/mol)**		
**Mn (modifier)**	1.577×10^{5} (±2.960%)	7.570×10^{4} (±2.661%)
**Mw (modifier)**	1.578×10^{5} (±2.956%)	7.570×10^{4} (±2.661%)
**Protein fraction moments**		
**Fw**	0.327 (±0.036%)	0.005 (±0.725%)
