# Supplementary figures and images for: Escherichia coli O127 group 4 capsule proteins assemble at the outer membrane
Source: PLoS One. 2021 Nov 15;16(11):e0259900. doi: 10.1371/journal.pone.0259900 (PMC8592465; doi:10.1371/journal.pone.0259900)

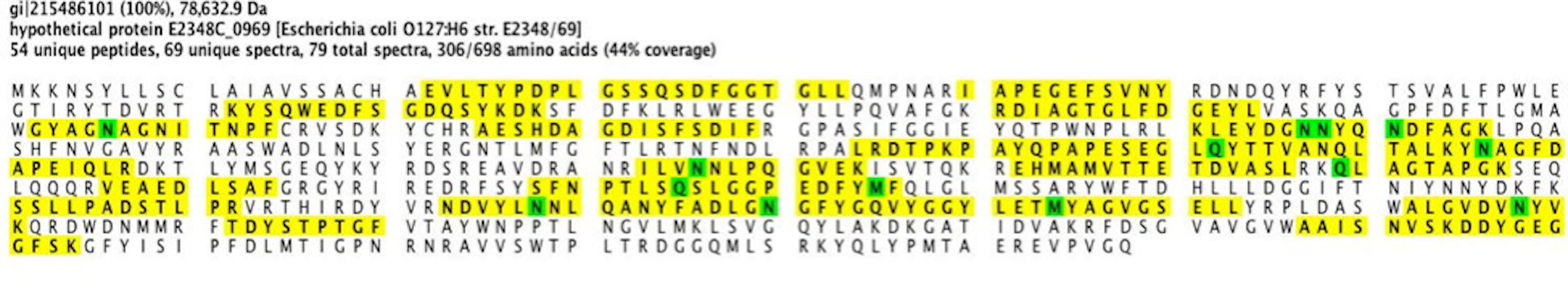

Supplement: S1 Fig — The first peptide sequence identified EVLTYP… by MS/MS was identical to that determined from N-terminal sequencing. (TIF) [file pone.0259900.s004.tif]

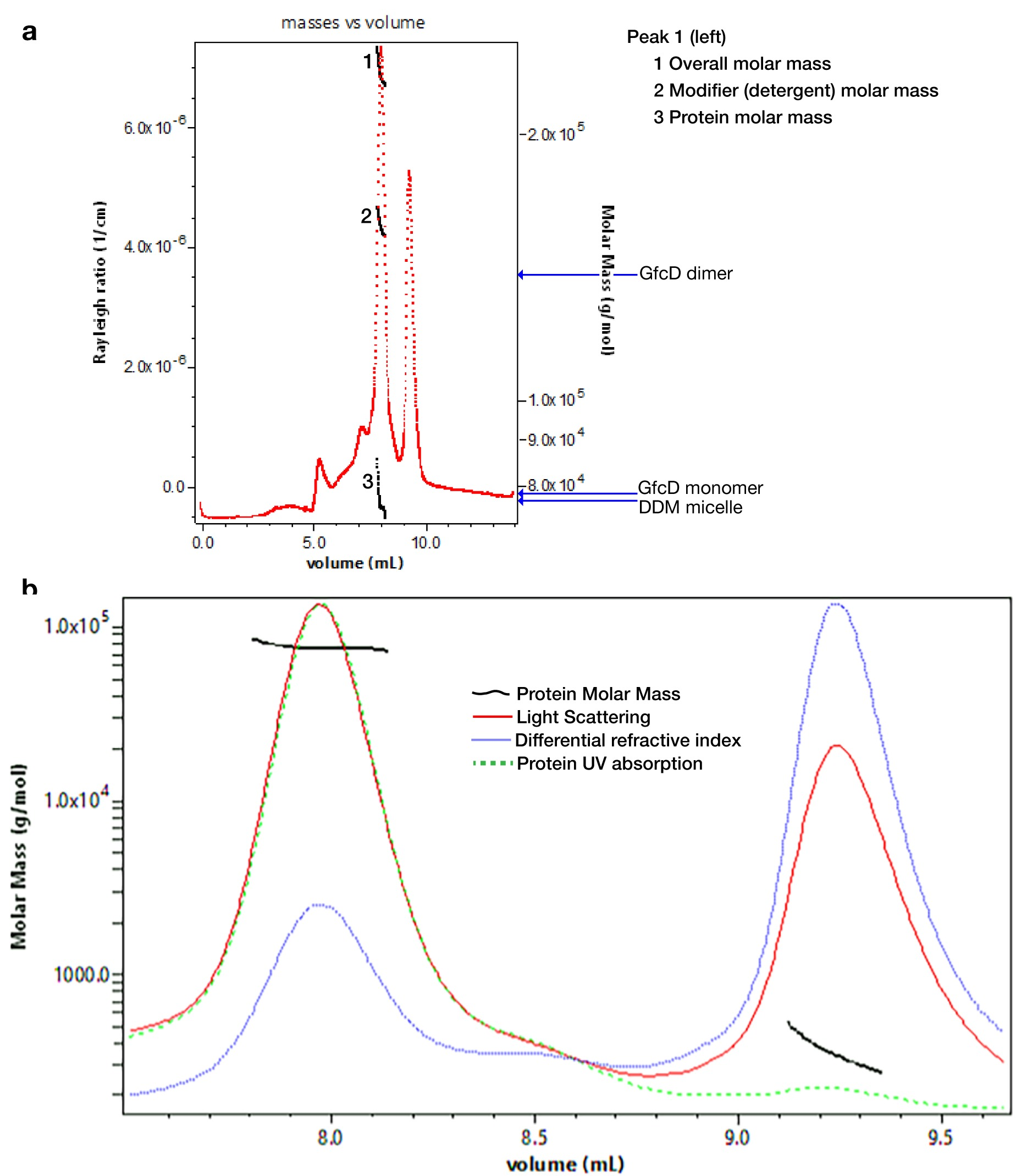

Supplement: S2 Fig — (a) The red trace is the light scattering shown on the left Y-axis. The numbers identified in the legend indicate molecular masses (right Y-axis) calculated by protein conjugate analysis of the MALS data for peak 1 (S3 Table). Fifty μL of protein was applied to a WTC-030S5 SEC silica column and eluted with 14 mL of buffer at a flow rate of 0.4 mL per minute. Other details in Experimental procedures. (b) Similar representation showing light scattering (red), differential refractive index (blue), and protein absorption curves (dashed green). Protein molar masses are shown in black. Only the first peak contains a protein mass consistent with GfcD. The second peak is primarily DDM micelles. (TIF) [file pone.0259900.s005.tif]

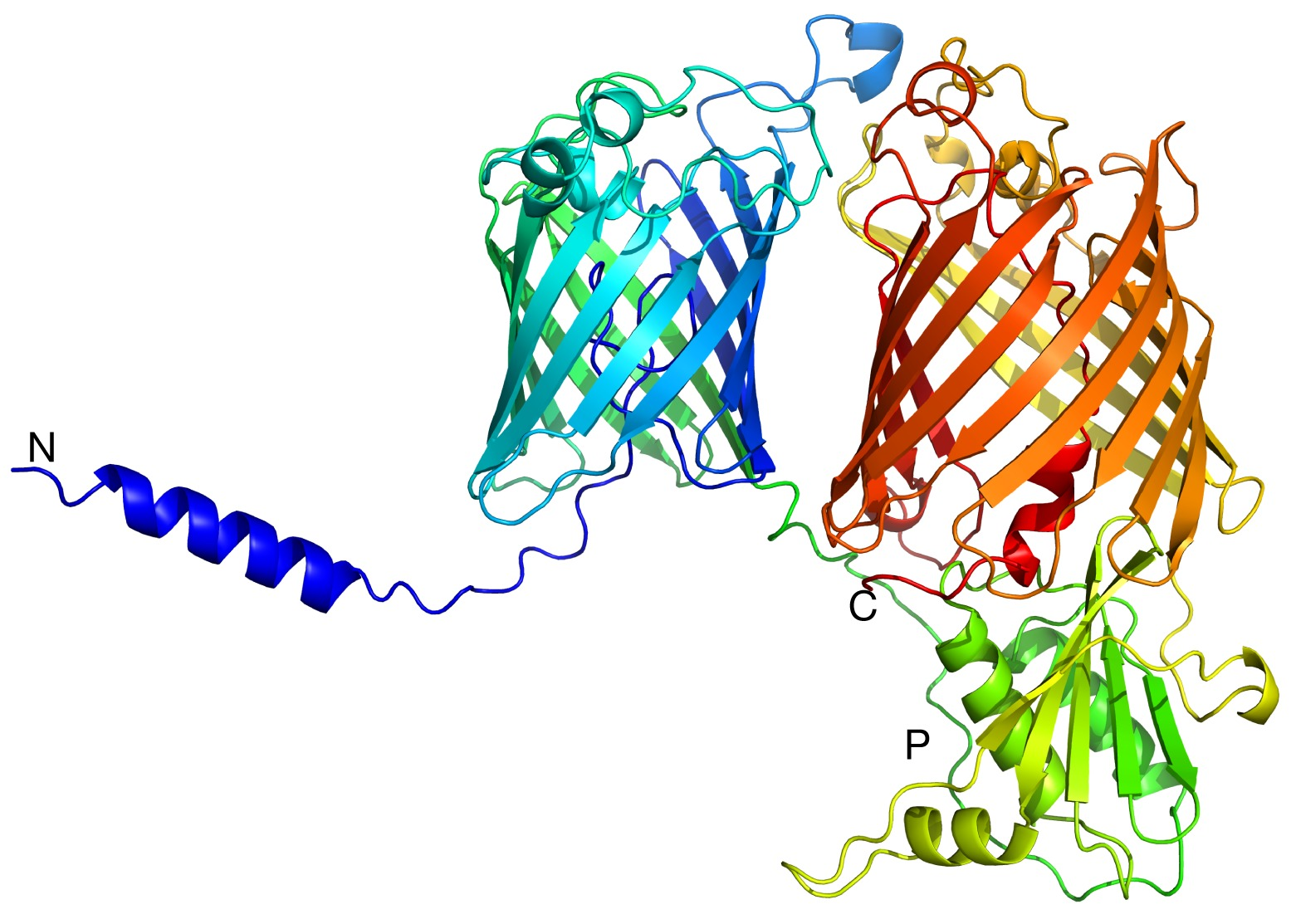

Supplement: S3 Fig — (TIF) [file pone.0259900.s006.tif]
