# Supplementary material for: Escherichia coli O127 group 4 capsule proteins assemble at the outer membrane
Source: PLoS One. 2021 Nov 15;16(11):e0259900. doi: 10.1371/journal.pone.0259900 (PMC8592465; doi:10.1371/journal.pone.0259900)

S1 File. GfcB and GfcC protein purifications; GfcD SEC profiles in detergents

GfcB Protein Purification

The cell strain *E. coli* Tuner(DE3)/pLacI with pMCSG7(*gfcB*) plasmid was grown in Luria Broth until it reached OD600 = 1.9 at which point expression of GfcB was induced with 1m M IPTG and the culture temperature was lowered to 30° C.

The lysate of the cells was initially passed over a TALON NiNTA column, and the elution of GfcB occurred near 40% of the 300 mM imidazole gradient.

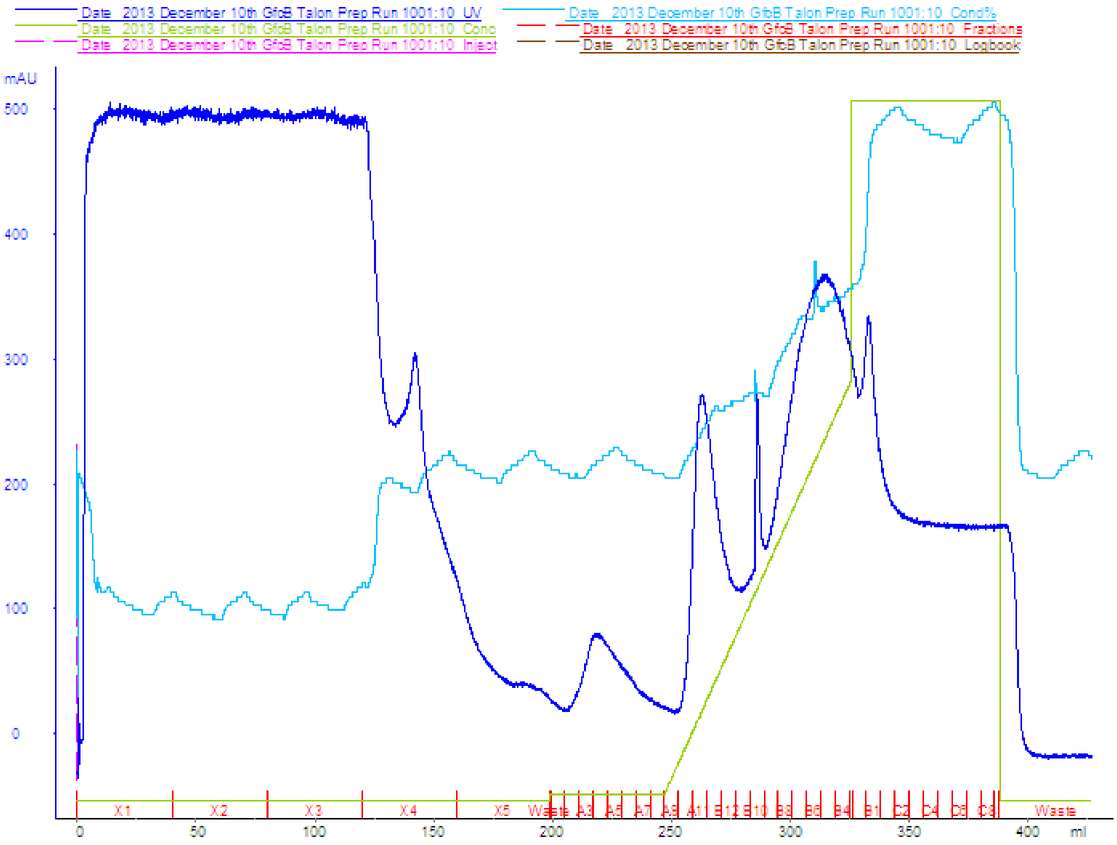

Lane	Sample (gel 1)	Lane	Sample (gel 2)
1	Benchmark Ladder	1	B6
2	Load	2	B4
3	X1	3	B2
4	X4	4	Benchmark Ladder
5	A1	5	B1
6	A4	6	C1 *
7	A7	7	C3
8	A9	8	C5
9	A11	9	C7
10	A12	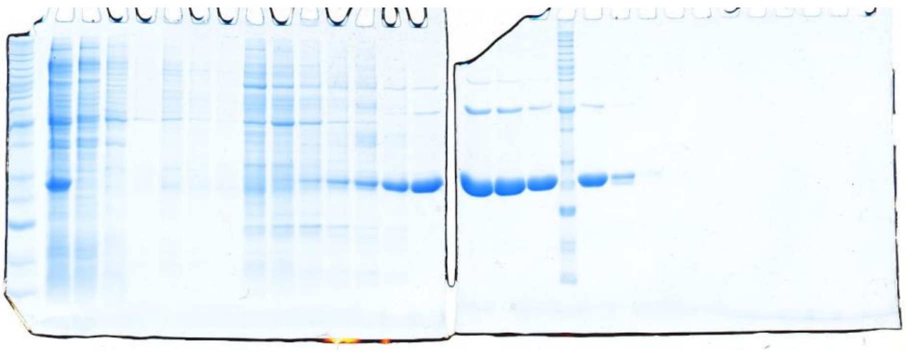	
11	B12		
12	B11		
13	B10		
14	B9 *		
15	B8		

The elution peak largely contained GfcB. The major pool of B9-C1 was combined and dialyzed into50 mM NaPO4 pH 7.4, 150 mM NaCl, 0.5 mM EDTA, and 1 mM DTT and treated with TeV protease to remove the poly-histidine tag. The protein was then dialyzed into 50mM sodium phosphate pH 7.4, 300mM NaCl, 5mM Imidazole and was passed again over the TALON NiNTA column (next page).

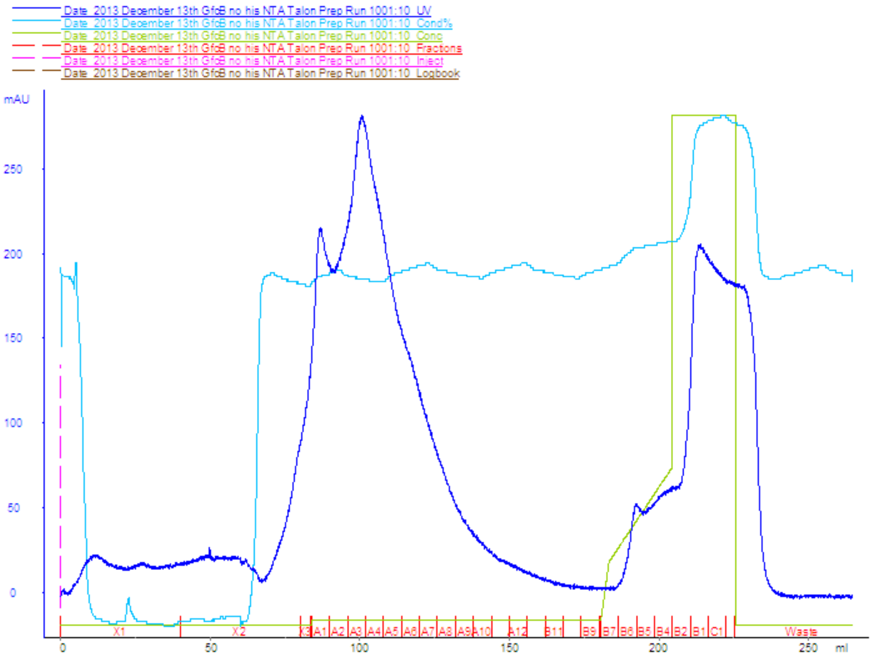

Lane	Sample
1	Benchmark Ladder
2	Before TeV
3	Post TeV
4	X1
5	X2
6	A2
7	A4
8	A6
9	A8
10	A12
11	B10
12	B5
13	B3
14	B1
15	C1

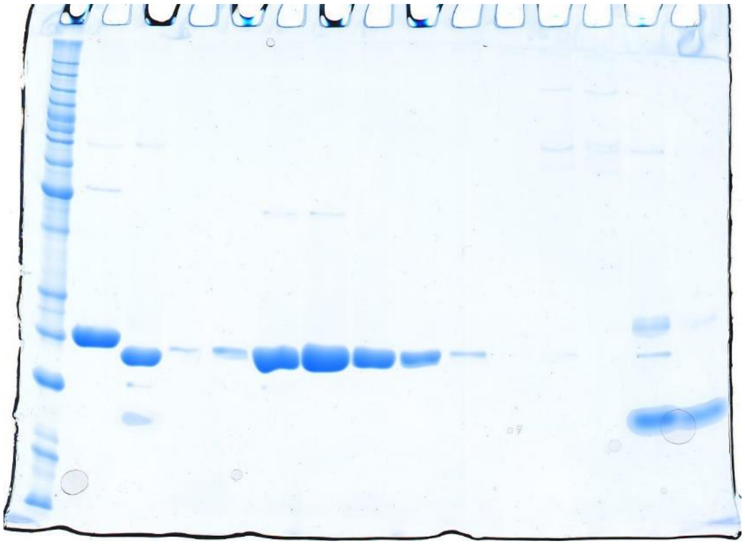

The flow-through fractions (X3, A1-A9) contained the GfcB protein without His-tag or TeV protease and were combined. The estimated purity based on the SDS-PAGE gels was greater than 95%.

GfcC Protein Purification

The cell strain *E. coli* Tuner(DE3)/pMCSG7(gfcC) was grown in Terrific Broth with Ampicillin and Chloramphenicol until the OD600 reached 0.8, then moved to 22° C and induced with IPTG.

The cells were harvested after 4 hours and the cell pellet dissolved in 50 mM sodium phosphate pH 7.4, 300 mM NaCl, 5 mM Imidazole and protease inhibitor tablet. The cells ere sonicated for 5 minutes at 60% intensity while keeping temperatures below 10 degrees Celsius.

The lysate was filtered and passed over a TALON NiNTA affinity column.

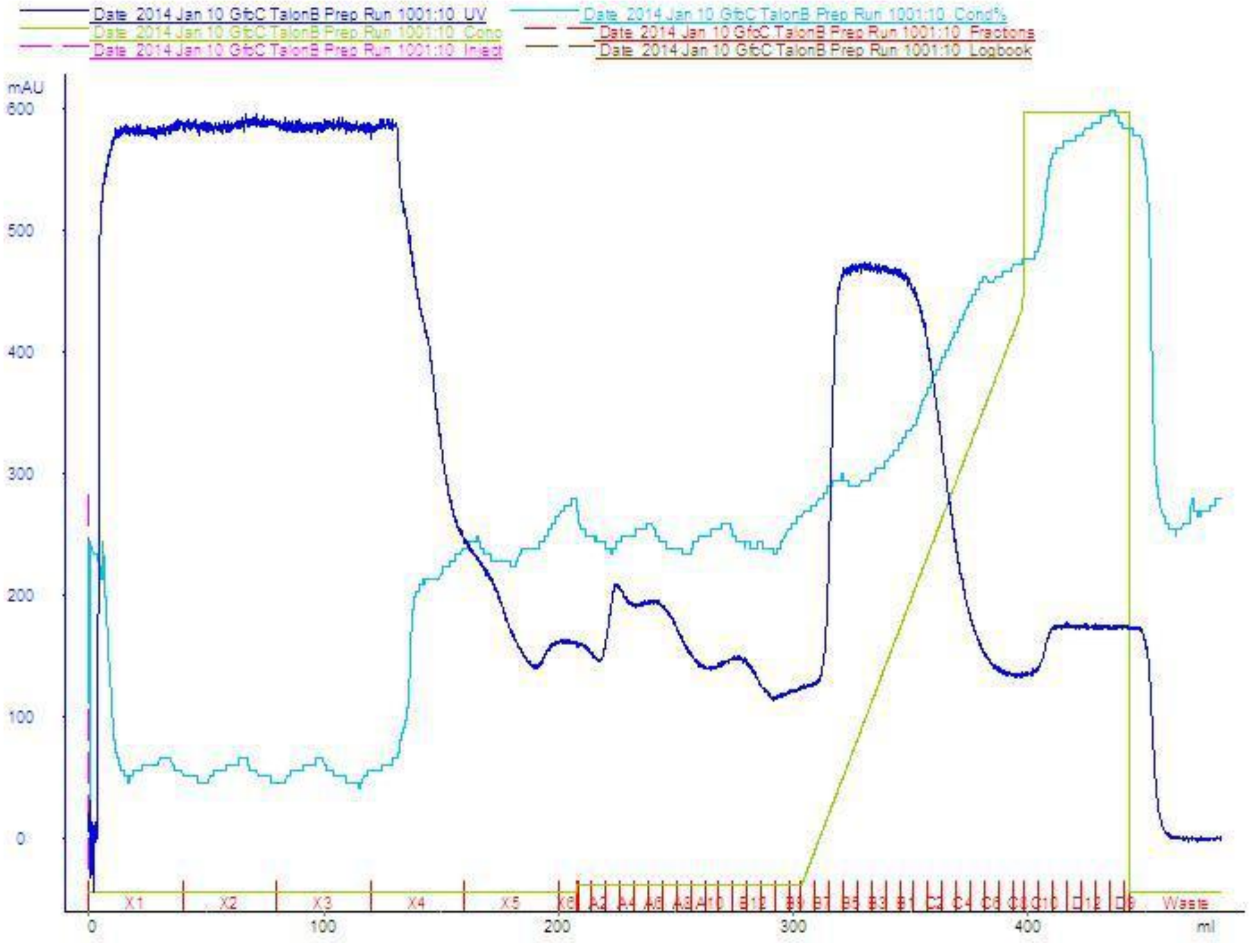

The pool of B9-C5 fractions were dialyzed overnight 4°C in 2 liters of 50 mM NaPO4 pH 7.4, 150 mM NaCl, 0.5 mM EDTA, and 1 mM DTT, and then 2 mL of TeV protease was added to cleave the His-tags.

Afterwards the protein was dialyzed in 50 mM NaPO4 pH 7.4, 300 mM NaCl, 5 mM Imidazole, and then passed over the Talon NiNTA column again (next page), this time collecting the flowthrough (protein without polyhistidine tag) as the target.

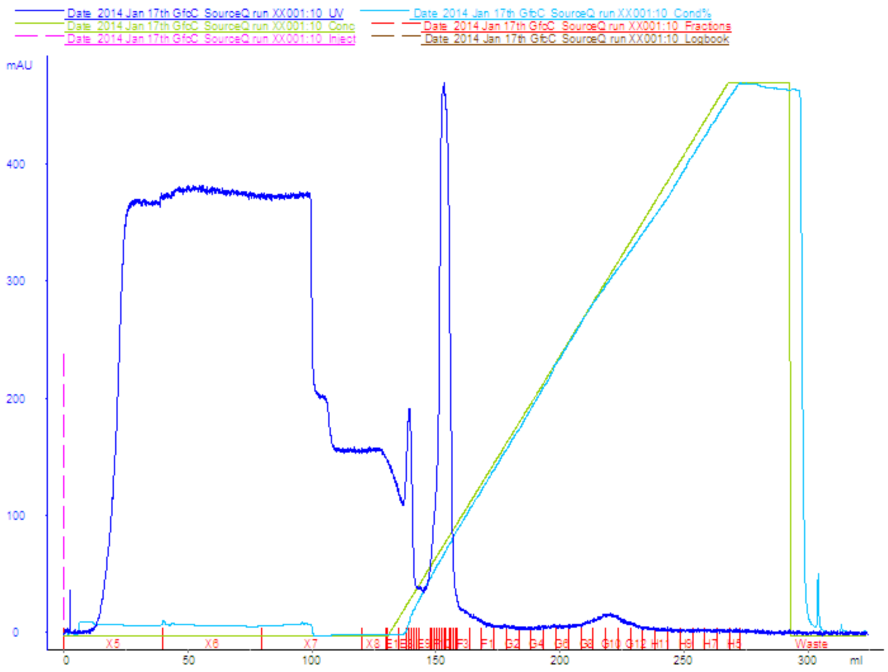

Lane	Sample
1	Benchmark ladder
2	Before TeV
3	After 2 day TeV
4	X6
5	X7
6	X8
7	E5
8	E7
9	E9
10	E11
11	F11
12	F9
13	F7
14	F5
15	G10

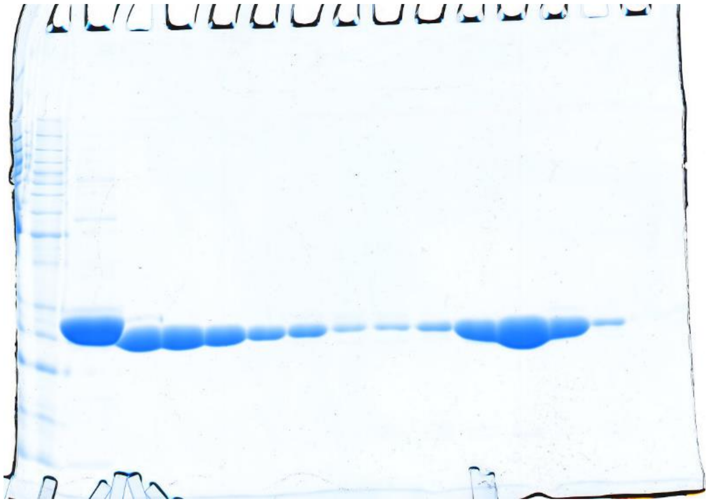

The purified GfcC protein demonstrated a shift in molecular weight after the cleavage with the TeV protease (lane 2 vs lane 3). Fractions X5–X8 (lanes 4–6) were pooled and concentrated. The overall purity of the final protein was estimated > 95% based on the lack of other bands in the SDS-PAGE gel.

GfcD in exchanged different detergents

The purified GfcD protein was exchanged into different detergents including *N,N*-dimethyl-n-dodecylamine N-oxide (LDAO), decylglucoside (DG), and dodedecylmaltoside (DDM). The most symmetrical elution profile was obtained with GfcD in the presence of DDM. Detergents creating smaller micelle sizes such as DG and LDAO resulted in less symmetrical peaks in the size exclusion chromatography.

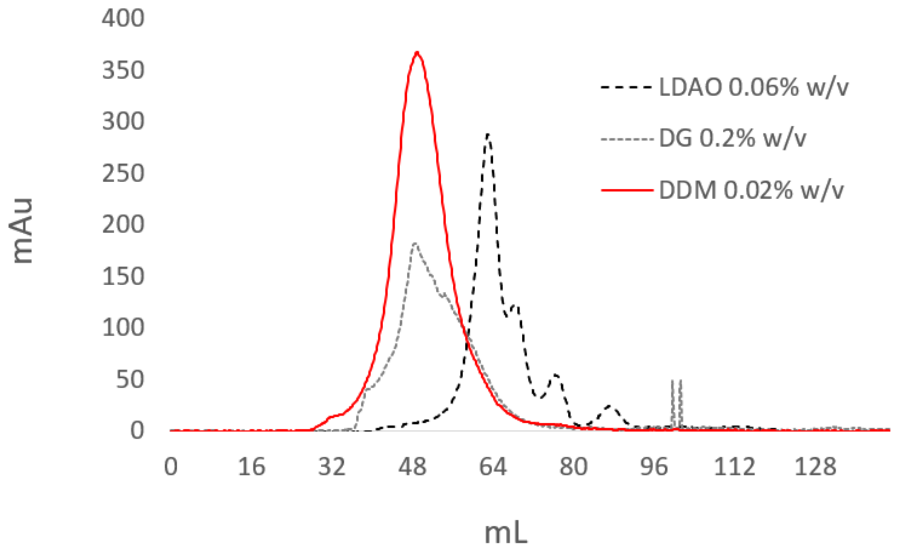

Supplement: S1 File — (PDF) [file pone.0259900.s007.pdf]
