# Supplementary material for: Escherichia coli O127 group 4 capsule proteins assemble at the outer membrane
Source: PLoS One. 2021 Nov 15;16(11):e0259900. doi: 10.1371/journal.pone.0259900 (PMC8592465; doi:10.1371/journal.pone.0259900)
Supplement: S1 Raw images — (PDF) [file pone.0259900.s008.pdf]

Fig3b

Each of these blots is against the same series of fractions (tubes) taken across the sucrose gradient fractionation (see actual figure). All blots contain the same samples in lanes as described below. Etk is predicted to be an 82-kDa molecular weight.

1 hour blocking at room temperature using 5% Milk in TBST
For anti-His blot:
anti-his 1:3500 in 10mL of 1% Milk in TBST for 1.5 hours at RT
3X washes with 10mL of TBST
anti-mouse-HRP 1:5000 in 10mL of 1% milk in TBST at 4C overnight rocking.
3X washes with 10mL of TBST

For anti-OmpA blot:
anti-ompA 1:3500 in 10mL of 1% Milk in TBST for 1.5 hours at RT
3X washes with 10mL of TBST
anti-rabbit-HRP 1:5000 in 10mL of 1% milk in TBST at 4C overnight rocking
3X washes with 10mL of TBST

For anti-Etk blot:
Anti-Etk 1:3500 in 1% Milk / TBST for 1.5 hours at RT
3X washes with 10mL of TBST
anti-rabbit-HRP 1:5000 in 10mL of 1% milk in TBST at 4C overnight rocking.
3X washes with 10mL of TBST

Pierce ECL kit to develep, 1:1mL and let sit briefly.

Lane	Sample (EPEC at 30C)
1	Supersignal 4uL (see below)
2	Tube 1
3	2
4	3
5	4
6	5
7	6
8	7
9	8
10	9
11	10

12	11
13	12
14	13
15	Control pMCSG26-GfcD

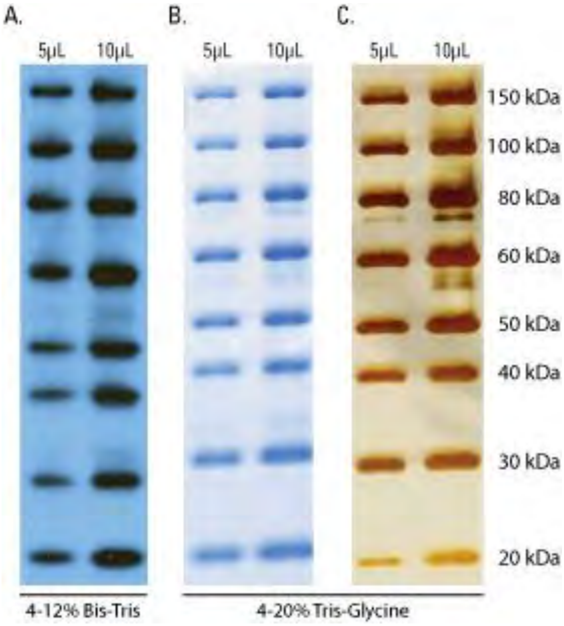

Reference SuperSignal protein ladder from the vendor manual, describing how it should appear on a blot.

Fig 3b, first row
anti-His (GfcD-His)

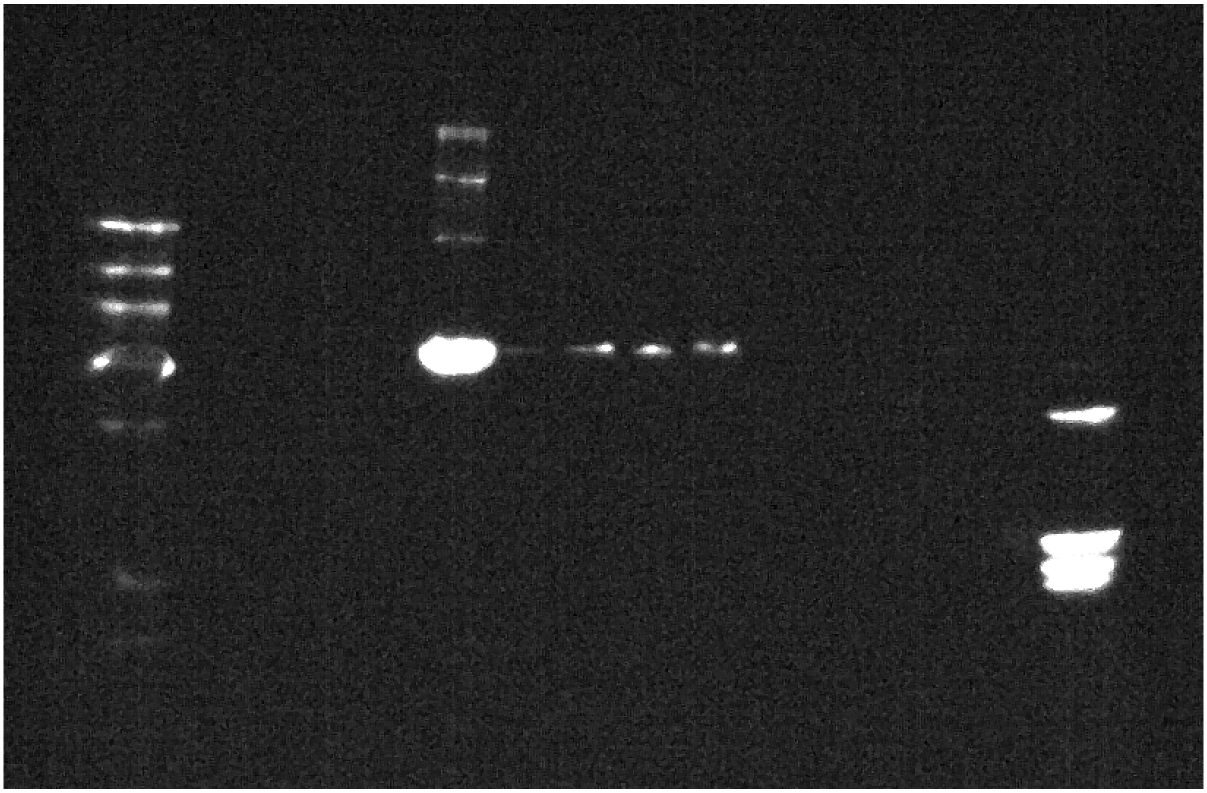

1 2 3 4 5 6 7 8 9 10 11 12 13 14 15
X

Fig 3b, second row
anti-OmpA

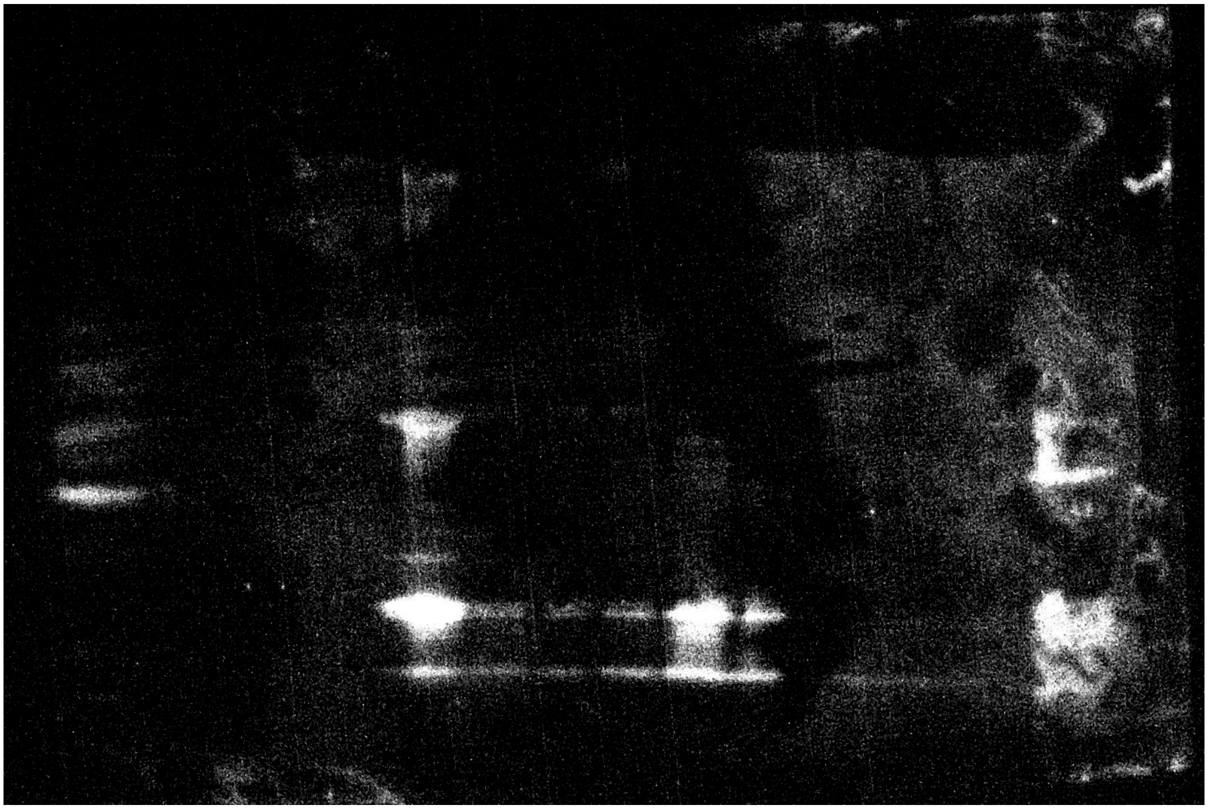

1 2 3 4 5 6 7 8 9 10 11 12 13 14 15
X

Fig 3b, third row
anti-Etk

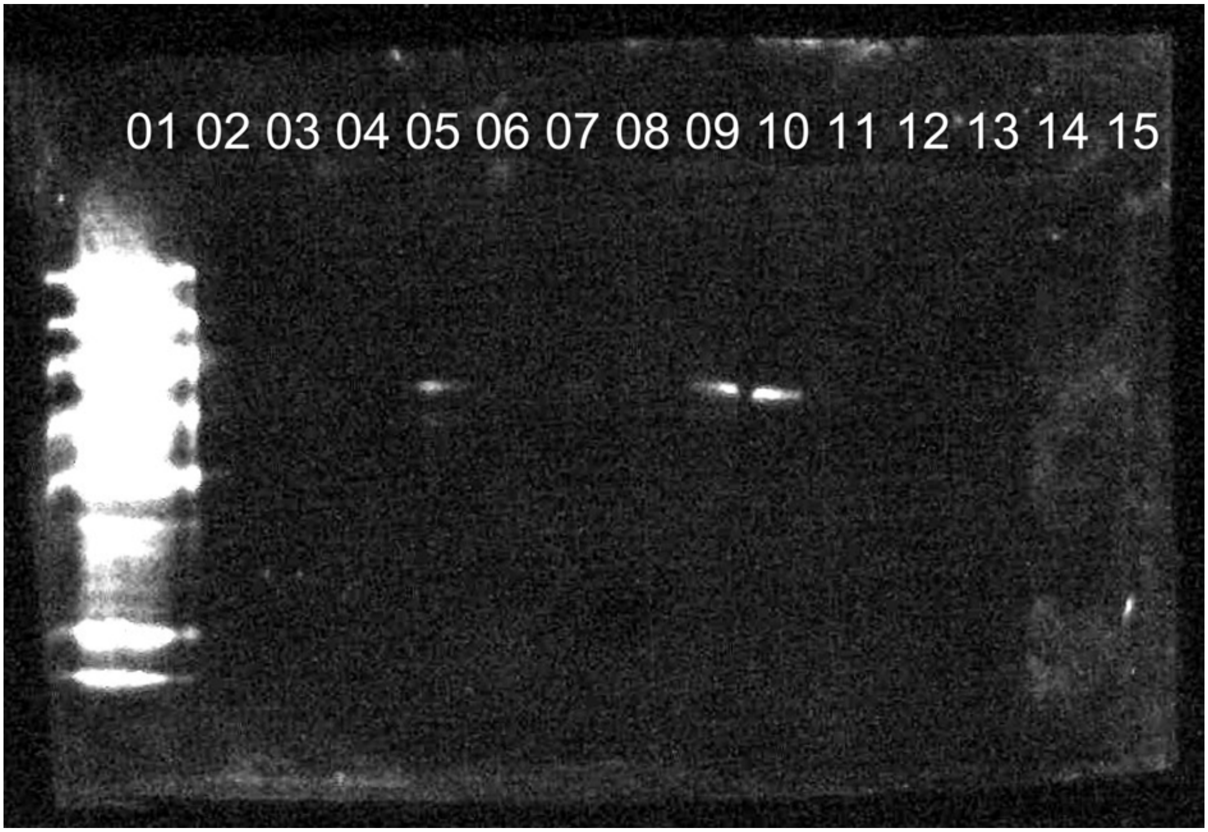

Fig 3c: Pulldown experiment using polyhistidine-tagged GfcB protein to pulldown and elute GfcC and GfcD proteins.

a. Prepare buffers:
    a. A: 50mM NaPO4 pH 7.4, 300mM NaCl, 0.1% DDM
    b. B : 50mM NaPO4 pH 7.4, 300mM NaCl, 10mM Imidazole, 0.1% DDM
    c. C: 2X Sample buffer containing + 50mM EDTA (elution buffer).
b. Wash and prepare the HisPur NiNTA spin column (200uL of Thermo-Scientific).
c. Separate the resin into 3 x 40uL aliquots in 1.5mL eppendorf tubes. Need 6 total tubes.
d. Add 150uL of the detergent solubilized protein to the tops of the resin, and let incubate for 30 minutes. Save at least 20uL for gels if possible.
e. After incubation, remove and begin 2x washes with 200uL of buffer A (15 column volumes). Then, 2x washes with 200uL of buffer B. Each wash, spin down 1 minute at 5000 RPM, then remove the supernatant.
f. Elute the protein using buffer 80uL buffer C. Do this by mixing with the resin, and incubating for 10 minutes with rocking, then spin down. I let sit with the resin for 30 min at 4C, and when I looked after sitting for a while the resin was more whiteish, possibly it takes a while to release the Ni from the resin
g. Boil the samples and load a 15% SDS-PAGE gel to do blotting.

Control experiment (right side of actual figure), with GfcB, GfcC, and GfcD (all without poly-histidine tags). 3 original blots "BCD-XX" (shown below table)

Lane	Sample
1	BCD M
2	BCD W
3	BCD E
4	Mix of<br>GfcB-his (25)<br>GfcC-his(27)<br>GfcD-His (76)
5	BCD DSP M
6	BCD DSP W
7	BCD DSP E
8	BCD DC4 M
9	BCD DC4 W
10	BCD DC4 E

Fig 3c
BCD (control) blots from right side of final figure

anti-GfcD

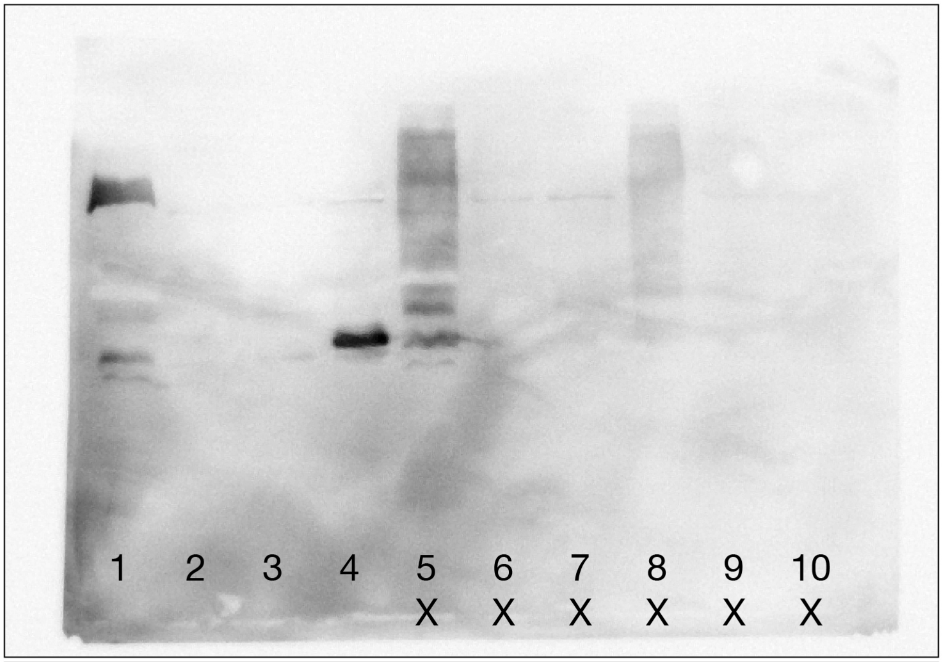

anti-GfcC

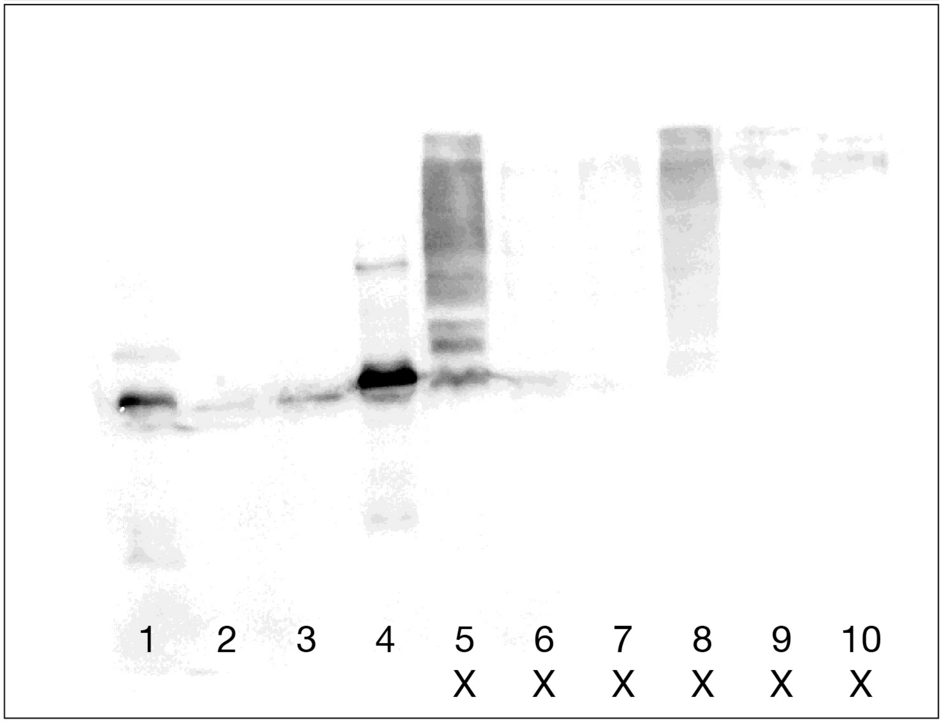

anti-His (His-GfcB)

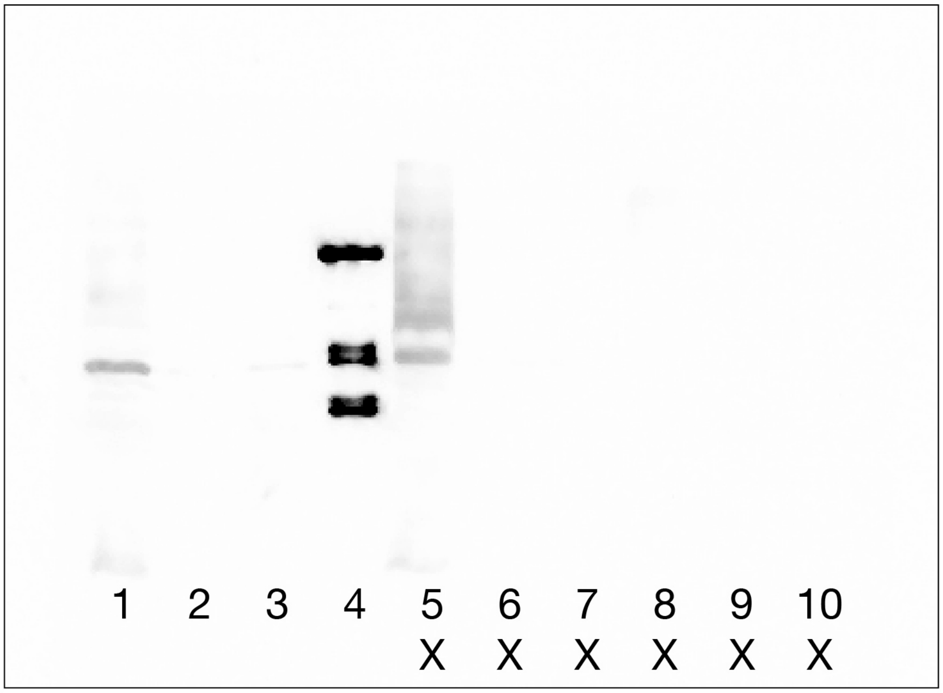

Pulldown using GfcB-polyhistine tag protein to pull down GfcC and GfcD. Blots labeled "BhCD-XX".

Lane	Sample
1	BhCD M
2	BhCD W
3	BhCD E
4	BhCD DSP M
5	BhCD DSP W
6	BhCD DSP E
7	Mix of<br>GfcB-his (25)<br>GfcC-his(27)<br>GfcD-His (76)
8	BhCD DC4 M
9	BhCD DC4 W
10	BhCD DC4 E

Fig 3c
B-his-CD blots
from left side of final figure

anti-GfcD

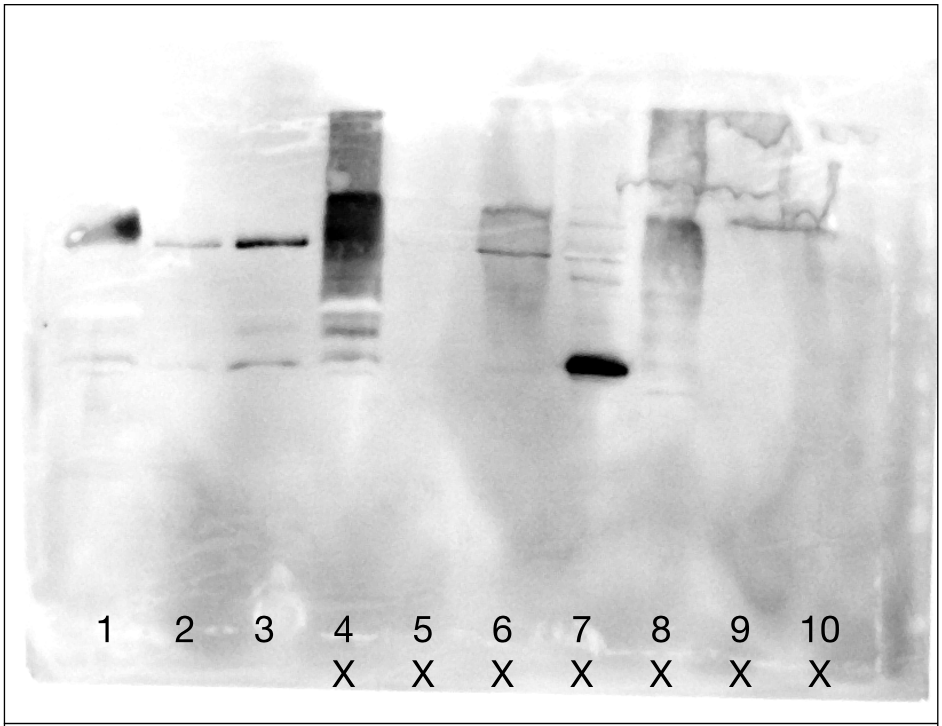

anti-GfcC

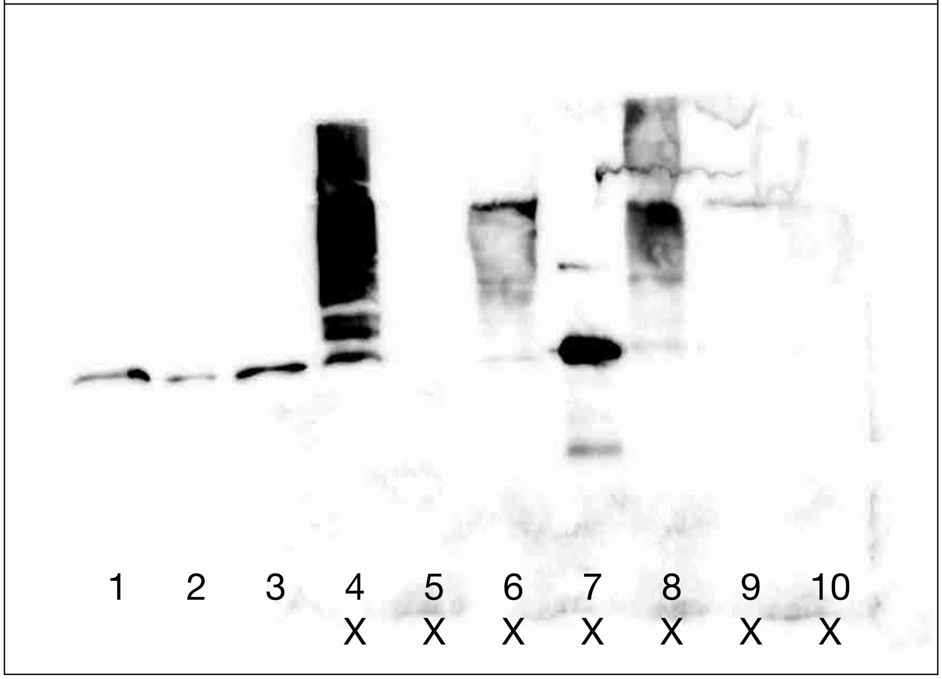

anti-His (His-GfcB)

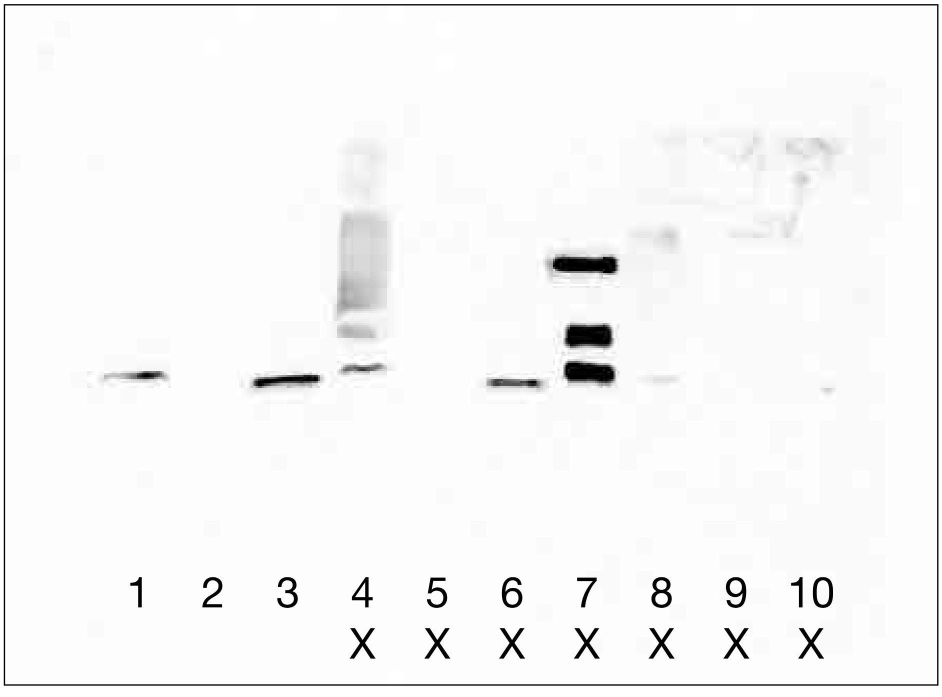

Fig 3e:

We have a cropped image that shows lanes 3 - WT / psa10(gfcD) and lanes 4 - WT / pSA10(gfcBCD) from the anti-GfcD blot.

8/16/2013 - blocked the blots overnight in 5% milk / TBST at 4C
Using 1:5000 in 10mL of new anti-GfcC antibody (rabbit PA6825, Day 35 bleed) put rest of 1mL vial at 4C in antibody box.
Using 1:5000 in 10mL of anti-GfcD, 2nd bleed.
Using 1:2000 of anti-His in 10mL.

All primary antibodies will incubate for 1.5 hours at 22C with shaking.
3X washes with 10mL of TBST
30 min with 1:5000 secondary anti-rabbit, or 1:2500 anti-mouse (for his-tag)
3X washes with 10mL of TBST

Lane	Sample
L	Ladder Supersignal 5uL
1	WT
2	gfcD:kan / pSA10(gfcD)
3	WT / pSA10(gfcD)
4	WT / pSA10(gfcBCD)
5	gfcC:kan / pSA10(gfcC)
6	WT / pSA10(gfcC)
7	WT / pSA10(gfc-B-his-CD)
8	" supernatant of culture
9	

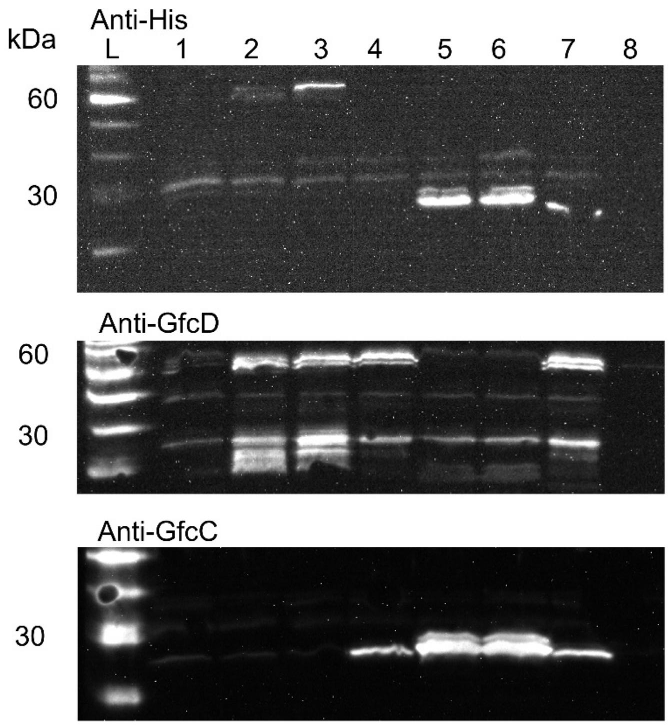

Figure 5a lane descriptions and information.

Lane	Sample
1	GfcD
2	GfcC
3	GfcB
4	GfcC+D
5	GfcB+D
6	GfcB+C
7	GfcB+C+D
8	Ladder (Benchmark or Supersignal)
9	GfcD*
10	GfcC*
11	GfcB*
12	GfcC+D*
13	GfcB+D*
14	GfcB+C*
15	GfcB+C+D*

Figure 5a.

(*) Lanes 9-15 are with DTT added to break crosslinks.

All the gels are 11% SDS-PAGE. The addition of DTT resulted in additional binding of the Anti-His or Anti-GfcC antibodies at lower molecular monomeric weights but did not eliminate binding at higher molecular weights. Binding of anti-His and anti-GfcC antibodies was only observed in lanes containing the appropriate target proteins (GfcB-his or GfcC).

GfcB has polyhistidine-tag. GfcC and GfcD are in absence of polyhistidine-tag.

Anti-His 1:2000 and Anti-GfcC at 1:5000 dilution.

We are finding staining of Anti-His at high weights with GfcB-his and GfcC in lanes 6 or 14.

We also see binding of GfcB-his with GfcD at higher molecular weights, see lanes 5, 7, and 13 and 15.

Fig 5a
SDS-PAGE (not in actual figure)

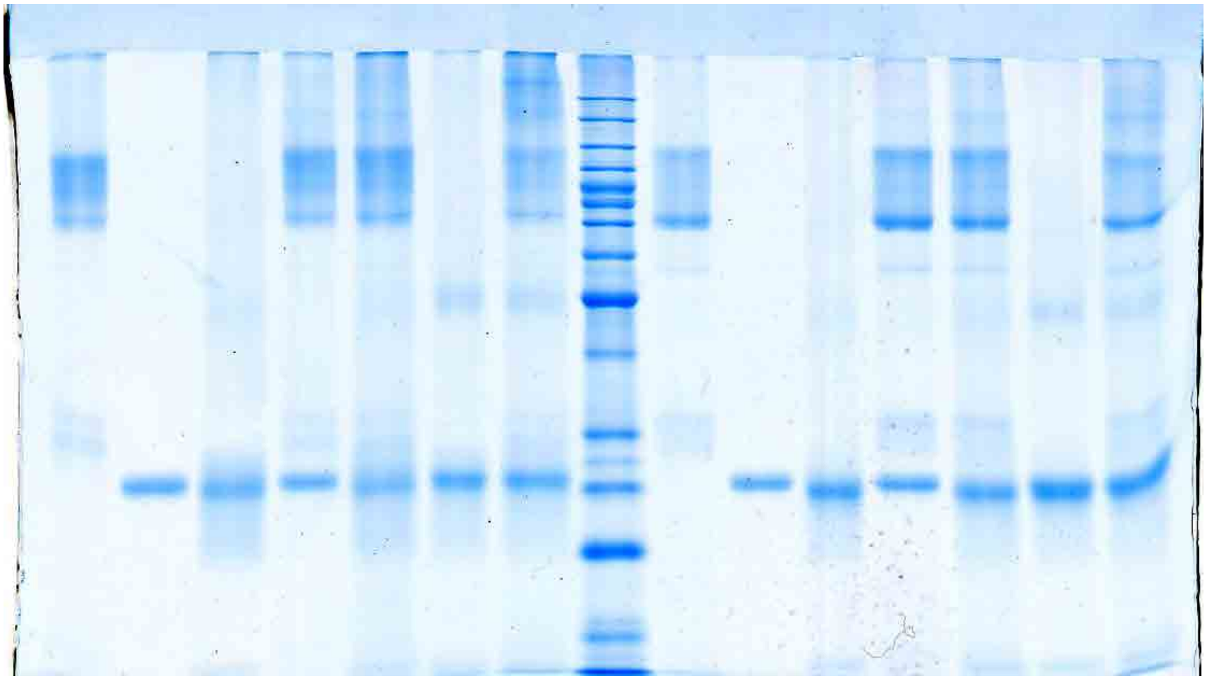

1 2 3 4 5 6 7 M X X X X X X X

Fig 5a
anti-GfcC original blot scan

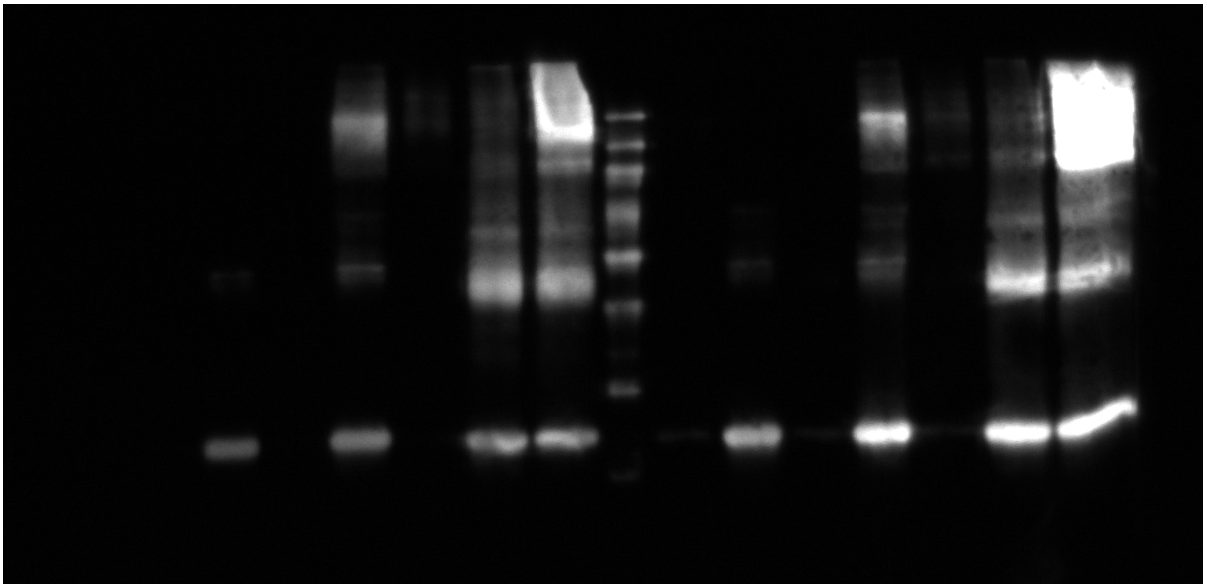

1 2 3 4 5 6 7 M X X X X X X X

Molecular weight markers are annotated in the figure in the paper.

Fig 5a
anti-His (GfcB-His)

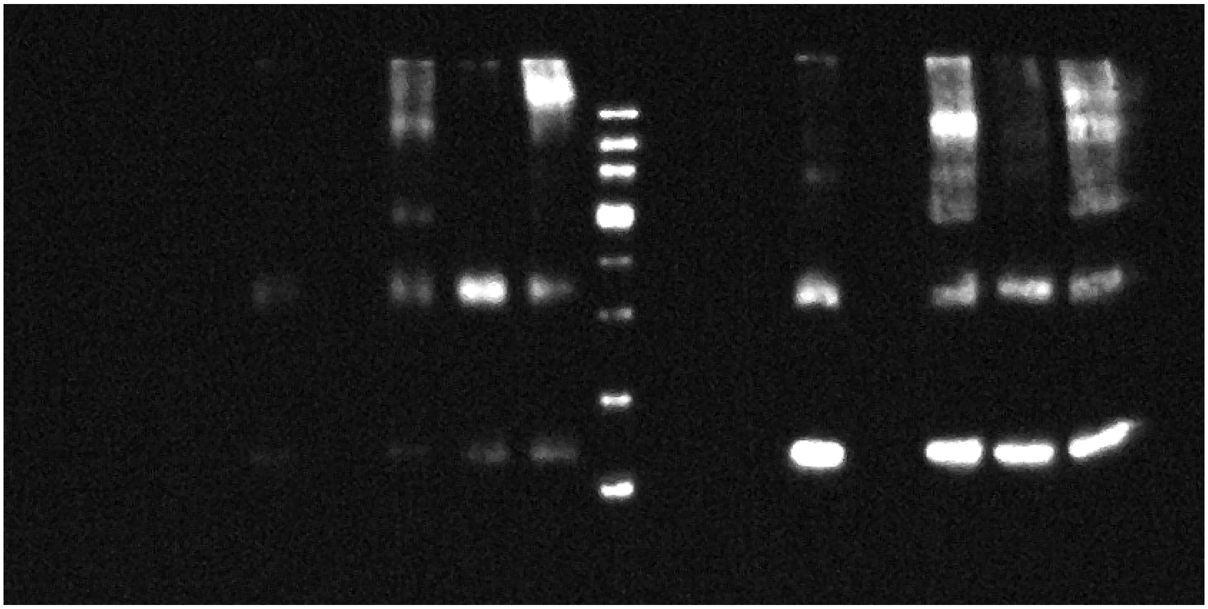

1 2 3 4 5 6 7 M X X X X X X X

Molecular weight markers are annotated in the figure in the paper.
